# Highly efficient one-step microwave-assisted synthesis of structurally diverse bis-substituted α-amino acid derived diimides†

**DOI:** 10.1039/c8ra05835k

**Published:** 2018-08-23

**Authors:** Marcin Konopka, Grzegorz Markiewicz, Artur R. Stefankiewicz

**Affiliations:** Faculty of Chemistry, Adam Mickiewicz University Umultowska 89b 61-614 Poznań Poland ars@amu.edu.pl; Center for Advanced Technologies, Adam Mickiewicz University Umultowska 89c 61-614 Poznań Poland

## Abstract

We report herein a facile and widely applicable microwave-assisted protocol for the synthesis of symmetrical diimides based on three structurally distinct aromatic dianhydrides: pyromellitic (PMA), biphenyl-tetracarboxylic acid (BPDA) and benzophenone-tetracarboxylic (BTDA) and five natural amino acids (Phe, Tyr, Ile, Lys, Cys). Fifteen symmetrical diimides with different structural characteristics containing a variety of functional groups can be produced with high yields and on a large scale.

## Abstract 2

We present a facile and widely applicable microwave-assisted protocol for the synthesis of symmetrical diimides based on three distinct aromatic dianhydrides and five natural amino acids.

## Graphical abstract

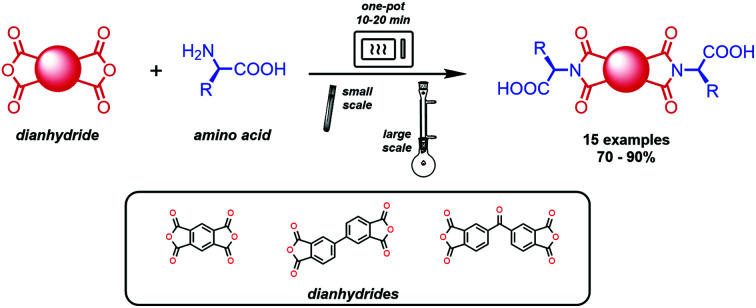

## Introduction

Symmetrical diimides have found a wide spectrum of uses in the last decade.^1^ The vast majority of these materials have structures based on naphthalene diimide (NDI) or perylene bisimide (PBI) cores.^1,2^ Both systems have constituted essential components of numerous non-covalent polymers^3,4^ and self-assembled supramolecular architectures,^5,6^ and have been used in DNA complexation^7–9^ and biomolecular recognition.^10,11^ Currently, there is an increased interest in this class of compounds, especially those bearing unique functional substituents and containing alternative central diimide cores, due to their significant application potential in materials chemistry and receptors in supramolecular chemistry.^12–14^ Despite the widely reported synthetic methodologies for the preparation of NDI and PBI derivatives, the literature protocols concerning compounds that are alternatives to them are scant.

It has been previously shown that α-amino acid derived NDIs can be obtained in high yields by microwave assisted synthesis.^15,16^ Although the protocols developed have considerably improved the preparation of these molecules in terms of time and ease of workup, the studies were devoted to a single group of diimides only *i.e.* NDIs, leaving the others to be synthesised by classical synthesis which was found to be dependent in an ill understood manner on the particular choice of dianhydride and amine. Thus, the synthesis of this important group of molecules is still associated with time-consuming and complicated protocols often leading to contaminated products in low yields.^17–19^ In addition, purification of these molecules is usually very difficult because of their generally low solubility and tendency to form undefined polymeric materials. For these reasons, the development of an accessible methodology based on microwave-assisted synthesis of non-NDI/PBI diimides in short reaction times, with easy work up and in high yields is greatly desirable from the viewpoints of both synthesis and the application potential of these molecules.

**Scheme 1:**
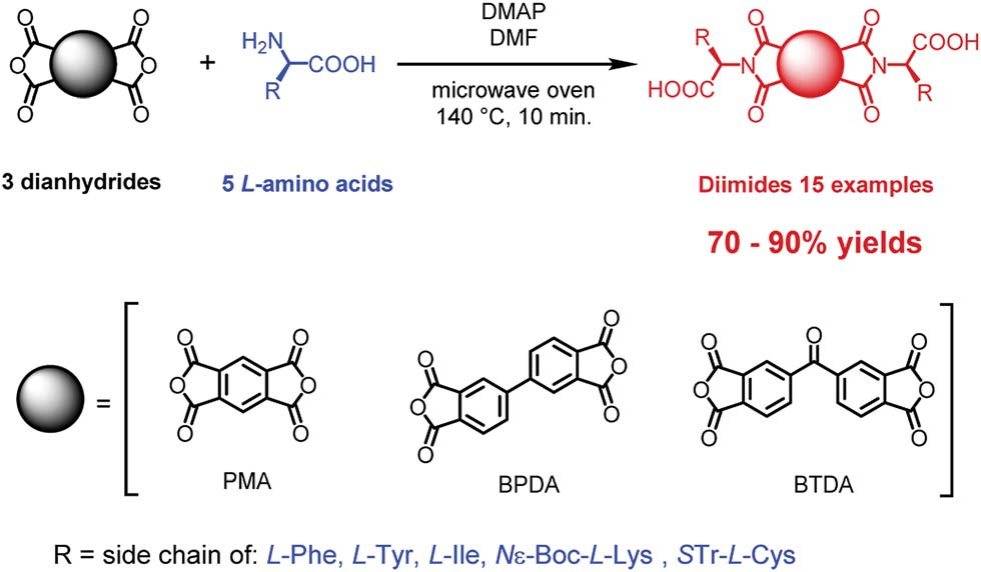
General synthetic method for the microwave assisted preparation of symmetrical diimides from three distinct dianhydrides and α-amino acids.

Here, we describe a one-step process for microwave assisted synthesis of symmetrical α-amino acid derived diimides based on three structurally distinct aromatic cores namely, pyromellitic dianhydride (PMA),^20^ biphenyl-tetracarboxylic acid dianhydride (BPDA)^21^ and benzophenone-tetracarboxylic acid dianhydride (BTDA)^22^ (Scheme 1). We chose these anhydrides due to their potential to deliver diimides with properties different to those of NDIs and PBIs. The PMA little core was found as an interesting platform for designing n-channel transistor semiconductors based on pyromellitic diimides.^23^ BPDA introduces a free rotation axis in the center of diimide molecule and this offers a means to engender of new class of symmetrical diimides with greater conformational lability. BTDA has a ketone group in the center of molecule which offers prospects for further modification or provision of an attachment point for coupling of two or more diimide molecules into larger systems.^24^ Use of well-known reversible reactions like imine formation, reductive amination or formation of hemithioacetals was seen as a pathway to further variations. All three aromatic platforms were functionalized with five different amino acids: l-phenylalanine, l-tyrosine, l-isoleucine, *N*ε-Boc-l-lysine and *S*-Tr-l-cysteine. These were selected as structurally diverse species providing a stringent test of the sensitivity of our methods to the presence of several functional groups. We have prepared fifteen diimides, nine of them new, in very high yields independent of the reaction scale, and without evidence of racemization at the chiral centers.

## Results and discussion

### General synthetic methodology

The enantiomerically pure diimides were synthesized as shown in Scheme 1. The reactions were conducted in pressure-resistant glass vessels with a total volume of ∼10 mL, designed for CEM microwave ovens and closed with silicone/PTFE caps.

The optimal scale for these reactions was to use 0.5 mmol of an anhydride, 1.0 mmol of amino acid, and ∼2 mmol of base, which is equal to less than 1 g of total material load. We found that higher loadings led to significant contamination with thermal decomposition products. The reaction mixtures were irradiated for 10 min at 140 °C using full microwave power (300 W) for the temperature ramping (∼30 s) followed by short pulses (20–40 W) to maintain the scheduled conditions. After cooling to room temperature, the reaction mixtures were poured into hydrochloric acid (1.0 mol × dm^−3^), the solid product was filtered off, washed with deionized water and dried in high vacuum for 12 h. This procedure turned out to be sufficient to obtain the desired products with high spectroscopic and analytical purity (See Experimental section for details).

## Method optimisation

We found that the procedure originally presented by Pantoş *et al.*^15,16^ for naphthalene diimides (NDI), which utilizes DMF as a solvent and triethylamine (Et_3_N) as a base catalyst, could also be used for the systems described here but the use of these reagents is associated with certain difficulties. Although DMF is perfectly suited for conducting nucleophilic reactions under microwave conditions, due to its high dipole moment and low vapor pressure, as well as its stability under microwave conditions, very often its residues are problematic during reaction work-up and purification of the products. Et_3_N, due to its very high volatility, generates high pressures (0.5–1.0 MPa) in the closed reaction systems, which limits its application only to expensive high-pressure reactors with fixed volume and synthesis scales. To facilitate reaction work-up and be able to carry out reaction on the larger scales at atmospheric pressure, we therefore investigated modification of this procedure. The reaction between PMA and l-phenylalanine was chosen as a model for the optimization of the synthetic methodology. These reagents give the diimide, PMI-Phe, in the form of very fine powder requiring only a simple water wash during workup. Two different solvents, *N*,*N*-dimethylformamide (DMF) and acetonitrile (MeCN), and three bases: triethylamine (Et_3_N), 4-(dimethylamino)pyridine) (DMAP), and 1,4-diazabicyclo[2.2.2]octane (DABCO) were selected for the test reactions.

The outcomes of the reactions performed under the different conditions are shown in Table 1. The highest yield (≈87%) was observed when DMF and DMAP were employed as solvent and base, respectively. Despite the fact that MeCN slightly reduces the reaction yields (down to ≈79% and ≈63% for the DABCO and DMAP, respectively), it is an interesting alternative for DMF, especially when fully aliphatic amino acids are used (see ahead). Complete removal of DMF from the products was possible through a combination of several water rinses, ultrasonic irradiation and pumping in vacuum, but in some cases, it was a very time-consuming process. The previously proposed pre-evaporation of the DMF^15,16^ from the reaction mixtures seems to be helpful but it does not solve the trace contamination problem. What we have now found is that the use of acetonitrile instead of DMF greatly facilitates the work-up, and significantly improves the separation and drying processes.

**Table 1:** Comparison between the reaction yields under different solvent/base conditions for representative reaction between PMA and l-Phe

Entry^a^	Base	Solvent	Reaction time^b^ (min)	Temp. (°C)	Yield^c^ (%)
1	Et_3_N	DMF	10	140	71–75
2	Et_3_N	MeCN	10	140	72–74
3	DABCO	DMF	10	140	51–56
4	DABCO	MeCN	10	140	77–81
5	DMAP	DMF	10	140	85–88
6	DMAP	MeCN	10	140	61–65

a Reactions were carried out on 0.5 mmol of dianhydride, 1.0 mmol of amino acid, 2.0 mmol of base and 5 mL of the solvent.

b Excluding temperature ramping.

c Each reaction was repeated 3-times, giving isolation yields in the ranges shown.

**Fig. 1:**
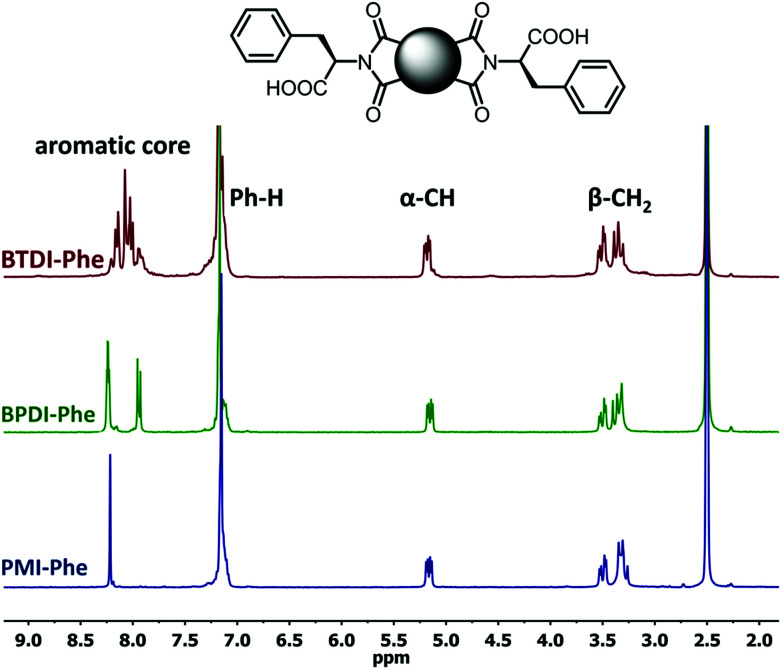
^1^H NMR (300 MHz, DMSO-*d*_6_) spectra of PMI-Phe (blue), BPDI (green), BTDI-Phe (red). Peaks description: α-CH (2H), β-CH_2_ (4H) and Ph-H (10H) stands from l-phenylalanine moieties.

The poorest results were obtained when DMF/DABCO and MeCN/Et_3_N pairs were employed. While the first pair is inefficient and gives contaminated products, the second generates high overpressure, which is problematic even for a dedicated microwave reactor. On the basis of these initial observations, we decided to synthesize the entire series of symmetrically functionalised diimides using the DMF/DMAP pair, since this gave the highest yield for the model reaction. As shown in Table 2, all fifteen different diimides were obtained in high yields and purities, indicating the broad utility of the procedure and thus that it should be useful for the synthesis of any desired diimide. We have not found any marked dependence on the structure of the substrates used and consider that the minor variations in yield are probably due to differences in solvation. In Fig. 1 we present the stacked ^1^H-NMR spectra of molecules PMI-Phe (blue), BPDI-Phe (green), BTDI-Phe (red) with l-phenylalanine and three structurally different dianhydrides. Signals due to the α-CH and β-CH_2_ protons of the amino acid residue appear at essentially identical positions (≈5.2 and 3.4 ppm, respectively).

**Table 2:** List of synthesized symmetrical diimides with structures and abbreviations

Entry	Dianhydride	Amino acid	Product^a^	Abbreviation	Reaction time^b^ (min)	Reaction temp. (°C)	Yield^c^ (%)
1	PMA	l-Phe	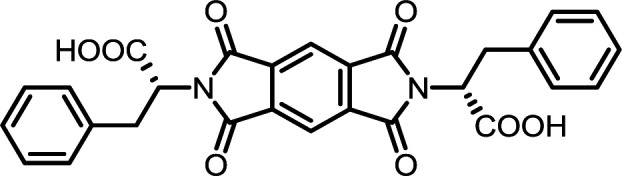	PMI-Phe	10	140	87
2	PMA	l-Tyr	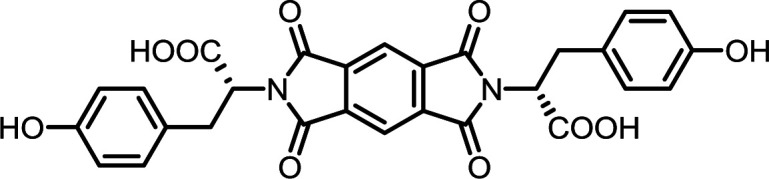	PMI-Tyr	10	140	87
3	PMA	l-Ile	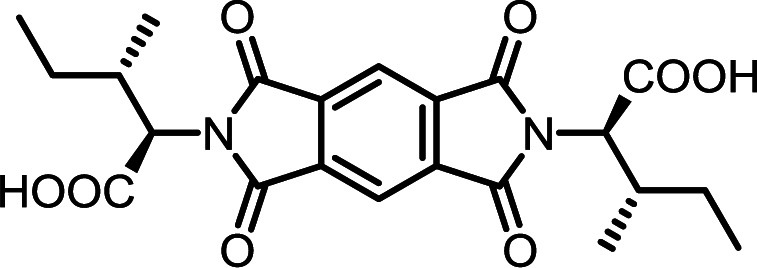	PMI-Ile	10	140	72
4	PMA	*S*-Tr-l-Cys	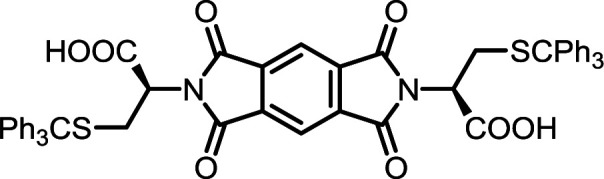	PMI-Cys	10	140	75
5	PMA	*N*ε-Boc-l-Lys	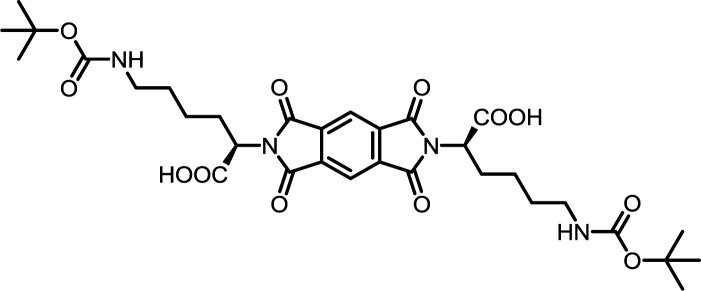	PMI-Lys	10	140	70
6	BPDA	l-Phe	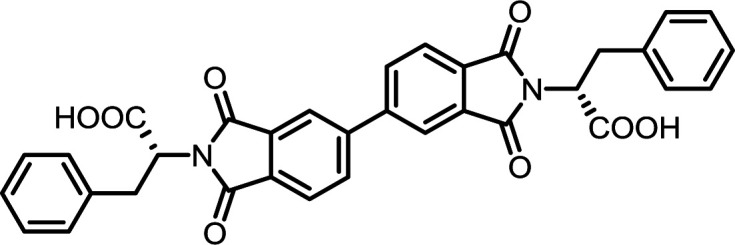	BPDI-Phe	10	140	84
7	BPDA	l-Tyr	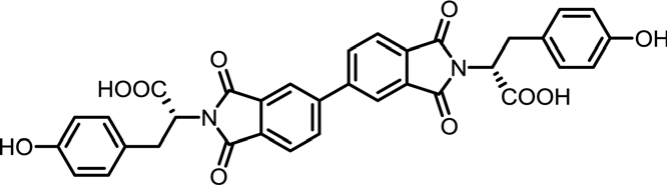	BPDI-Tyr	10	140	72
8	BPDA	l-Ile	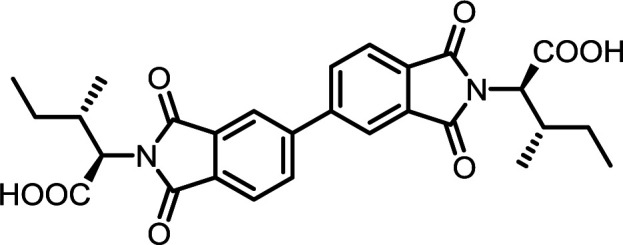	BPDI-Ile	10	140	72
9	BPDA	*S*-Tr-l-Cys	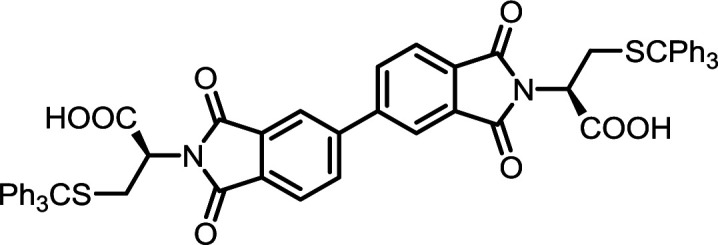	BPDI-Cys	10	140	90
10	BPDA	*N*ε-Boc-l-Lys	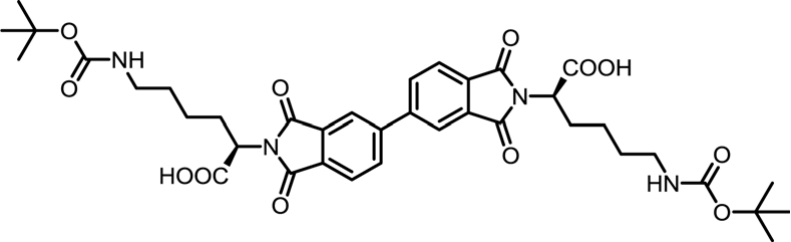	BPDI-Lys	10	140	75
11	BTDA	l-Phe	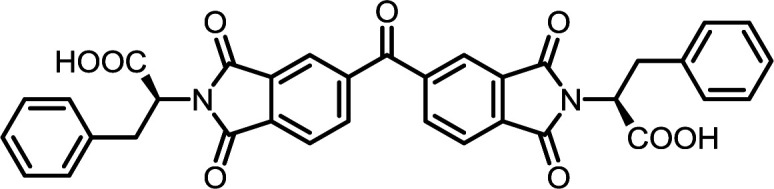	BTDI-Phe	10	140	75
12	BTDA	l-Tyr	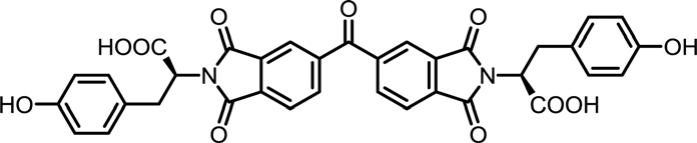	BTDI-Tyr	10	140	76
13	BTDA	l-Ile	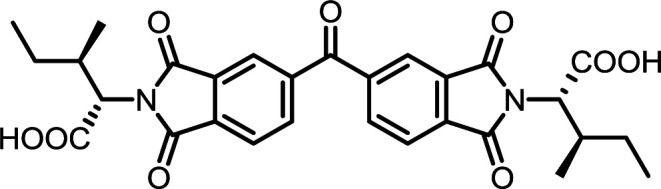	BTDI-Ile	10	140	79
14	BTDA	*S*-Tr-l-Cys	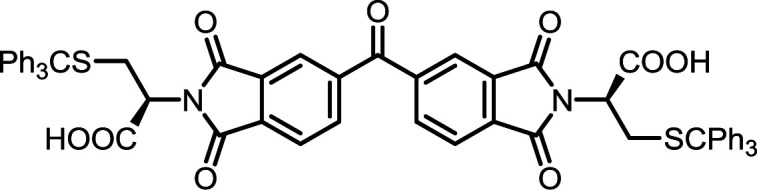	BTDI-Cys	10	140	90
15	BTDA	*N*ε-Boc-l-Lys	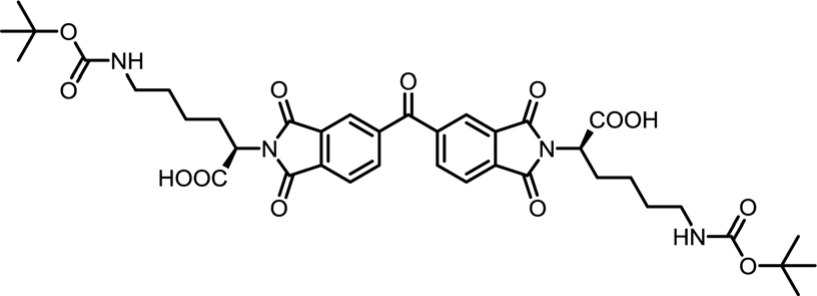	BTDI-Lys	10	140	70

a Reactions were carried out on 0.5 mmol of dianhydride, 1.0 mmol of amino acid and 2.0 mmol of DMAP in 5 mL of DMF.

b Excluding temperature ramping.

c Yield of isolated product.

Importantly, it was previously found that racemization of amino acid may occur when the fusion with anhydride is conducted at high temperature.^25^ In the present case, due to the presence of two chiral centers, it is possible to generate three products with *LL*, *DD* and *DL* configurations. However, the high symmetry of signals in the ^1^H NMR spectra, namely single set of resonances for α-CH and β-CH_2_ protons implies that the applied methodology leads to the formation of enantiomerically pure products. The only noticeable but obvious difference between the three spectra is the distribution of aromatic signals from central aromatic cores.

**Fig. 2:**
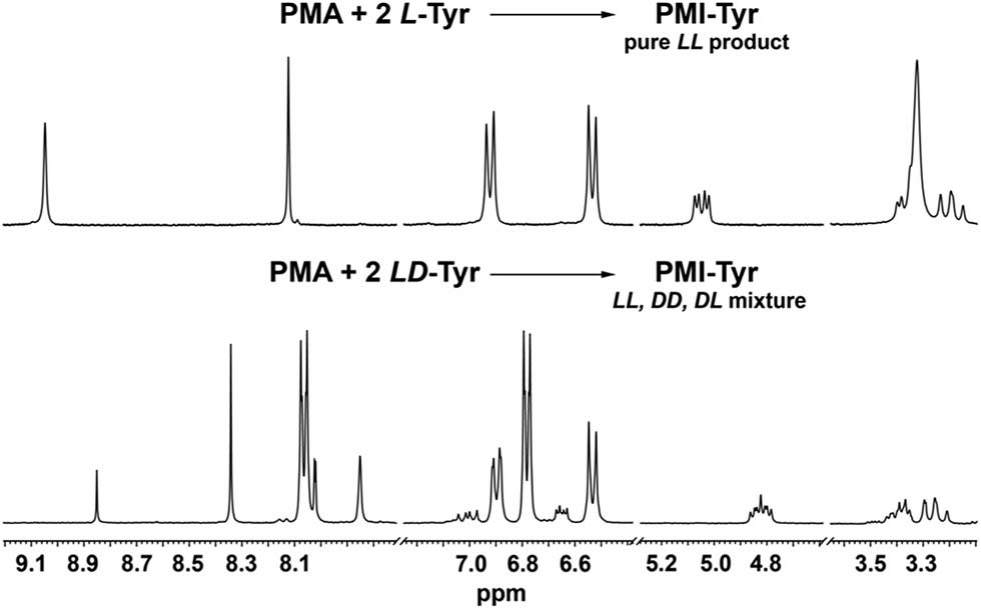
^1^H NMR (300 MHz, DMSO-*d*_6_) spectra of the products isolated after reaction of PMA dianhydride with chirally pure l-tyrosine (top) and racemic tyrosine (bottom).

To further confirm this, we performed the test reaction between PMA dianhydride and racemic tyrosine. Indeed as expected, the use of the substrate in its racemic form has led to a mixture of diastereomeric products whose presence was manifested by the multiplication of all resonances in the ^1^H NMR spectrum, in contrast to the fully symmetrical spectrum observed when chirally pure amino acids were used (Fig. 2).

### Large scale synthesis

The synthesis of symmetrical diimides is usually the first step in the synthesis of functionalized monomers used in self-assembled systems such as dynamers,^26^ so relatively large quantities of these precursors are often required. Therefore, we investigated whether the method above might be applied in reaction at a larger scale while maintaining the high yields and purities obtained in test reactions.

The high scale reactions were performed using 10.0 mmol of appropriate amino acid, and 5.0 mmol of dianhydride. The non-pressurized DMF/DMAP system was found to be well suited for the open vessel conditions. Reactions were conducted in a standard 100 mL round-bottom flask with an air-cooled condenser to provide reflux. Using the standard reaction times (10 min) the expected products were obtained in essentially identical yields compared to that observed in low scale under pressurized conditions. We were able to obtain up to 5 g of pure diimide in one large-scale batch and use it for further modification. The MeCN/DABCO system was also found to be useful in open-vessel reactions but due to the low boiling point of acetonitrile (82 °C), it was necessary to extend the reaction time up to 20 minutes in order to obtain yields comparable to that obtained under high pressure. The results of six large scale syntheses are shown in Table 3.

**Table 3:** Large scale synthesis results

Entry	Product^a^	Solvent	Base	Reaction time^b^ (min)	Reaction temp. (°C)	Yield^e^ (%)
1	PMI-Phe	DMF	DMAP	10	145^c^	88
2	PMI-Ile	MeCN	DABCO	20	82^d^	70
3	BPDI-Phe	DMF	DMAP	10	145^c^	82
4	BPDI-Ile	DMF	DMAP	10	145^c^	71
5	BTDI-Phe	DMF	DMAP	10	145^c^	73
6	BTDI-Ile	MeCN	DABCO	20	82^d^	75

a Reactions were carried out on 5.0 mmol of dianhydride, 10.0 mmol of amino acid and 20.0 mmol of base in 50 mL of given solvent.

b At constant microwave power = 180 W.

c ±5 °C maintained at constant power.

d Boiling point of the solvent.

e Yield of isolated product.

By conducting reactions at lower temperature (82 °C, 10 min) in the MeCN/DABCO mixture we were able to observe reaction intermediates, among which the most interesting are monosubstitution products that are essential in the synthesis of unsymmetrical bis-substituted compounds. Unfortunately, despite the utilisation of both thermodynamic and stoichiometric control, we were not able to isolate them using methodology presented in this work, as their high solubility in all range of solvents prevents their effective precipitation from reaction mixtures. Although it was possible to separate the monosubstituted products by reverse phase HPLC methods, we believe that easier and more efficient synthetic and isolation protocols may be developed which will open the way for the desymmetrisation of the presented diimides in the future.

## Experimental

### General considerations

All chemicals and solvents were purchased from commercial sources and used as received. NMR solvents were purchased from Deutero GmbH (Germany). NMR spectra were acquired on Bruker Fourier 300 spectrometer equipped with 1H/13C 5 mm probe, at 298 K and were referenced on solvent residual peaks. Mass spectra were recorded on Bruker Impact HD Q-TOF spectrometer in negative ion mode. Elemental analyses were obtained using an Elemental Analyser Vario EL III. Samples were dried for 12 h under high vacuum prior to analysis. The microwave assisted reactions were performed on CEM Discover SP oven.

### General method for the synthesis of symmetrical diimides

A representative example is that of the PMA and l-phenylalanine reaction. l-Phenylalanine (165.2 mg, 1.0 mmol), PMA (109.0 mg, 0.5 mmol) and DMAP (244.0 mg, 2.0 mmol) were placed in a glass vessel and suspended in 5 mL of dry DMF. The mixture was sonicated until complete dissolution had occurred. The vessel was sealed with a silicon cap and placed in a microwave oven. The mixture was heated at 140 °C for 10 min. After cooling, the mixture was poured into 50 mL of hydrochloric acid (1 mol × dm^−3^). The precipitate was filtered off and washed with 200 mL of water to remove residual DMF. The solid residue was dried under high vacuum. Yield: 223 mg (87%) of PMI-Phe.

### General method for the large-scale synthesis of symmetrical diimides

A representative example again is that of the PMA/l-phenylalanine reaction. PMA (1.09 g, 5.0 mmol), l-phenylalanine (1.65 g, 10.0 mmol) and DMAP (2.44 g, 20.0 mmol) were placed in 100 mL round-bottom flask and dissolved in 50 mL of dry DMF. The solution was heated in the microwave oven at 180 W for 10 min under open vessel conditions with an air condenser. The resulting solution was concentrated to a 10 mL volume on a rotary evaporator before being mixed with 200 mL of hydrochloric acid (1 mol × dm^−3^). The white precipitate was filtered off, washed with 500 mL of water and dried under high vacuum. Yield 2.12 g (83%) of PMI-Phe.

#### PMI-Phe

^1^H NMR (300 MHz, DMSO-*d*_6_) *δ* 13.47 (s, 2H), 8.22 (s, 2H), 7.23–7.07 (m, 10H), 5.16 (dd, *J* = 11.3, 4.8 Hz, 2H), 3.50 (dd, *J* = 14.1, 4.8 Hz, 2H), 3.39–3.24 (m, 2H). ^13^C NMR (75 MHz, DMSO-*d*_6_) *δ* 169.61, 165.18, 137.11, 136.24, 128.75, 128.40, 126.66, 118.51, 53.60, 33.92. ESI-MS: *m*/*z* calculated for: [M − H]^−^ 511.1147, found: 511.1153. Elemental analysis calcd (%) for C_28_H_20_N_2_O_8_: C 65.62, H 3.93, N 5.47; found: C 65.75, H 3.82, N 5.52.

#### PMI-Tyr

^1^H NMR (300 MHz, DMSO-*d*_6_) *δ* 13.40 (s, 2H), 9.15 (s, 2H), 8.22 (s, 2H), 6.92 (d, *J* = 8.0 Hz, 4H), 6.53 (d, *J* = 7.9 Hz, 4H), 5.05 (dd, *J* = 11.6, 4.8 Hz, 2H), 3.43–3.29 (m, 2H), 3.25–3.13 (m, 2H). ^13^C NMR (75 MHz, DMSO-*d*_6_) *δ* 169.69, 165.21, 155.89, 136.26, 129.70, 127.05, 118.48, 115.23, 53.97, 33.07. ESI-MS: *m*/*z* calculated for: [M − H]^−^ 543.1045, found: 543.1048. Elemental analysis calcd (%) for C_28_H_20_N_2_O_10_: C 61.77, H 3.70, N 5.15; found: C 61.72, H 3.73, N 5.11.

#### PMI-Ile

^1^H NMR (300 MHz, DMSO-*d*_6_) *δ* 13.16 (s, 2H), 8.33 (s, 2H), 4.60 (d, *J* = 7.8 Hz, 2H), 2.45–2.31 (m, 2H), 1.50 (ddd, *J* = 13.4, 7.6, 3.6 Hz, 2H), 1.06 (d, *J* = 6.7 Hz, 6H), 1.03–0.92 (m, 2H), 0.81 (t, *J* = 7.3 Hz, 6H). ^13^C NMR (75 MHz, DMSO-*d*_6_) *δ* 169.46, 165.80, 136.54, 118.44, 57.02, 34.05, 25.28, 16.66, 10.93. ESI-MS: *m*/*z* calculated for: [M − H]^−^ 443.1460, found: 443.1461. Elemental analysis calcd (%) for C_22_H_24_N_2_O_8_: C 59.46, H 5.44, N 6.30; found: C 59.34, H 5.48, N 6.34.

#### PMI-Lys

^1^H NMR (300 MHz, DMSO-*d*_6_) *δ* 13.27 (s, 2H), 8.32 (s, 2H), 6.71 (t, *J* = 5.7 Hz, 2H), 4.80 (dd, *J* = 7.7 Hz, 2H), 2.84 (q, *J* = 6.4 Hz, 4H), 2.14–2.05 (m, 4H), 1.40–1.21 (m, 26H). ^13^C NMR (75 MHz, DMSO-*d*_6_) *δ* 170.15, 165.66, 155.55, 136.67, 118.31, 77.27, 52.34, 28.99, 28.18, 27.71, 23.06. ESI-MS: *m*/*z* calculated for: [M − H]^−^ 673.2726, found: 673.2737. Elemental analysis calcd (%) for C_32_H_42_N_4_O_12_: C 56.97, H 6.27, N 8.30; found: C 57.13, H 6.25, N 8.40.

#### PMI-Cys

^1^H NMR (600 MHz, DMSO-*d*_6_) *δ* 13.66 (s, 2H), 8.38–8.27 (m, 2H), 7.30–7.26 (m, 32H), 4.59 (ddp, *J* = 14.2, 9.2, 4.8 Hz, 2H), 3.11–3.03 (m, 2H), 3.01–2.94 (m, 2H). ^13^C NMR (151 MHz, DMSO) *δ* 168.52, 165.02, 143.75, 136.42, 129.03, 128.31, 128.12, 126.22, 66.66, 55.82, 39.52, 30.37. ESI-MS: *m*/*z* calculated for: [M − H]^−^ 907.2153, found: 907.2158. Elemental analysis calcd (%) for C_54_H_40_N_2_O_8_S_2_: C 71.35, H 4.44, N 3.08; found: C 71.44, H 4.73, N 3.11.

#### BPDI-Phe

^1^H NMR (300 MHz, DMSO-*d*_6_) *δ* 13.38 (s, 2H), 8.31–8.18 (m, 4H), 7.94 (d, *J* = 8.2 Hz, 2H), 7.22–7.08 (m, 10H), 5.15 (dd, *J* = 11.6, 4.8 Hz, 2H), 3.50 (dd, *J* = 14.1, 4.9 Hz, 2H), 3.42–3.35 (m, 2H). ^13^C NMR (75 MHz, DMSO-*d*_6_) *δ* 170.04, 166.68, 144.64, 137.30, 134.14, 131.70, 130.46, 128.74, 128.37, 126.61, 124.05, 122.61, 53.11, 33.96. ESI-MS: *m*/*z* calculated for: [M − H]^−^ 587.1460, found: 587.1465. Elemental analysis calcd (%) for C_34_H_24_N_2_O_8_: C 69.38, H 4.11, N 4.76; found: C 69.42, H 4.27, N 4.82.

#### BPDI-Tyr

^1^H NMR (300 MHz, DMSO-*d*_6_) *δ* 13.32 (s, 2H), 9.15 (s, 2H), 8.29–8.16 (m, 4H), 7.97–7.93 (m, 2H), 6.94 (d, *J* = 8.1 Hz, 4H), 6.55 (d, *J* = 8.1 Hz, 4H), 5.05 (dd, *J* = 11.6, 4.9 Hz, 2H), 3.37 (dd, *J* = 14.2, 4.9 Hz, 2H), 3.25 (dd, *J* = 14.2, 11.7 Hz, 2H). ^13^C NMR (75 MHz, DMSO) *δ* 170.16, 166.77, 155.89, 144.68, 134.14, 131.77, 130.53, 129.71, 127.27, 124.06, 122.62, 115.23, 53.44, 33.14. ESI-MS: *m*/*z* calculated for: [M − H]^−^ 619.1358, found: 619.1359. Elemental analysis calcd (%) for C_34_H_24_N_2_O_10_: C 65.81, H, 3.90, N 4.51; found: C 65.77, H 4.03, N 4.62.

#### BPDI-Ile

^1^H NMR (300 MHz, DMSO-*d*_6_) *δ* 13.07 (s, 2H), 8.33 (d, *J* = 8.3 Hz, 4H), 8.05 (d, *J* = 7.8 Hz, 2H), 4.55 (d, *J* = 8.1 Hz, 2H), 2.39 (t, *J* = 8.3 Hz, 2H), 1.49 (ddd, *J* = 13.5, 7.5, 3.5 Hz, 2H), 1.07 (d, *J* = 6.7 Hz, 6H), 1.04–0.94 (m, 2H), 0.81 (t, *J* = 7.3 Hz, 6H). ^13^C NMR (75 MHz, DMSO-*d*_6_) *δ* 169.87, 167.13, 144.77, 134.09, 132.05, 130.78, 124.19, 122.63, 56.56, 33.94, 16.85, 10.87. ESI-MS: *m*/*z* calculated for: [M − H]^−^ 519.1773, found: 519.1764. Elemental analysis calcd (%) for C_28_H_28_N_2_O_8_: C 64.61, H 5.42, N 5.38; found: C 64.67, H 5.73, N 5.31.

#### BPDI-Lys

^1^H NMR (300 MHz, DMSO-*d*_6_) *δ* 13.19 (s, 2H), 8.33 (d, *J* = 7.3 Hz, 4H), 8.04 (d, *J* = 8.0 Hz, 2H), 6.74 (d, *J* = 6.2 Hz, 2H), 4.76 (t, *J* = 7.8 Hz, 2H), 2.86 (q, *J* = 6.2 Hz, 4H), 2.20–2.02 (m, 4H), 1.30 (s, 26H). ^13^C NMR (75 MHz, DMSO) *δ* 170.53, 167.04, 155.56, 144.70, 133.98, 132.21, 130.93, 124.15, 122.52, 77.27, 51.91, 29.02, 28.97, 28.20, 27.80, 23.16. ESI-MS: *m*/*z* calculated for: [M − H]^−^ 749.3039, found: 749.3042. Elemental analysis calcd (%) for C_38_H_46_N_4_O_12_: C 60.79, H 6.18, N 7.46; found: C 60.73, H 6.23, N 7.51.

#### BPDI-Cys

^1^H NMR (600 MHz, DMSO-*d*_6_) *δ* 8.33 (dd, *J* = 24.6, 16.7 Hz, 4H), 8.04 (d, *J* = 7.7 Hz, 2H), 7.28–7.20 (m, 30H), 4.56 (dd, *J* = 11.4, 4.5 Hz, 2H), 3.09 (t, *J* = 12.9 Hz, 2H), 2.91 (dd, *J* = 13.1, 4.3 Hz, 2H). ^13^C NMR (151 MHz, DMSO) *δ* 168.86, 166.50, 143.97, 143.76, 129.02, 128.32, 128.10, 127.76, 127.50, 126.88, 126.22, 66.50, 55.77, 39.52, 30.59. ESI-MS: *m*/*z* calculated for: [M − H]^−^ 983.2466, found: 983.2485. Elemental analysis calcd (%) for C_60_H_44_N_2_O_8_S_2_: C 73.15, H 4.50, N 2.84; found: C 73.24, H 4.63, N 2.89.

#### BTDI-Phe

^1^H NMR (300 MHz, DMSO-*d*_6_) *δ* 13.49 (s, 2H), 8.24–8.10 (m, 3H), 8.06–7.97 (m, 3H), 7.18 (d, *J* = 4.2 Hz, 10H), 5.18 (dd, *J* = 11.6, 4.8 Hz, 2H), 3.51 (dd, *J* = 14.1, 5.0 Hz, 2H), 3.35 (dd, *J* = 14.0, 11.5 Hz, 3H). ^13^C NMR (75 MHz, DMSO-*d*_6_) *δ* 193.05, 169.86, 166.28, 141.85, 137.22, 136.35, 133.59, 130.86, 128.73, 128.38, 126.64, 124.05, 123.85, 53.28, 33.94. ESI-MS: *m*/*z* calculated for: [M − H]^−^ 615.1409, found: 615.1413. Elemental analysis calcd (%) for C_35_H_24_N_2_O_9_: C 68.18, H 3.92, N 4.54; found: C 68.33, H 3.83, N 4.61.

#### BTDI-Tyr

^1^H NMR (300 MHz, DMSO-*d*_6_) *δ* 13.43 (s, 2H), 9.17 (s, 2H), 8.19–8.00 (m, 6H), 6.94 (d, *J* = 8.2 Hz, 4H), 6.56 (d, *J* = 8.5 Hz, 4H), 5.08 (dd, *J* = 11.5, 4.9 Hz, 2H), 3.38 (dd, *J* = 14.2, 4.9 Hz, 2H), 3.24 (dd, *J* = 14.2, 11.6 Hz, 2H). ^13^C NMR (75 MHz, DMSO-*d*_6_) *δ* 193.09, 169.98, 166.34, 155.89, 141.86, 136.39, 133.67, 130.94, 129.69, 127.16, 124.07, 123.85, 115.23, 53.61, 33.10. ESI-MS: *m*/*z* calculated for: [M − H]^−^ 647.1307, found: 647.1309. Elemental analysis calcd (%) for C_35_H_24_N_2_O_11_: C 64.82, H 3.73, N 4.32; found: C 64.87, H 3.93, N 4.26.

#### BTDI-Ile

^1^H NMR (300 MHz, DMSO-*d*_6_) *δ* 13.12 (s, 2H), 8.26–8.09 (m, 6H), 4.57 (d, *J* = 8.0 Hz, 2H), 2.47–2.30 (m, 2H), 1.62–1.38 (m, 2H), 1.07 (d, *J* = 6.7 Hz, 6H), 1.02–0.86 (m, 2H), 0.81 (t, *J* = 7.3 Hz, 6H). ^13^C NMR (75 MHz, DMSO-*d*_6_) *δ* 193.30, 169.68, 166.75, 141.86, 136.24, 134.03, 131.26, 124.13, 123.93, 56.74, 33.99, 25.33, 16.75, 10.91. ESI-MS: *m*/*z* calculated for: [M − H]^−^ 547.1722, found: 547.1725. Elemental analysis calcd (%) for C_29_H_28_N_2_O_9_: C 63.50, H 5.15, N 5.11; found: C 63.57, H 5.25, N 5.24.

#### BTDI-Lys

^1^H NMR (300 MHz, DMSO-*d*_6_) *δ* 13.22 (s, 2H), 8.27–7.97 (m, 6H), 6.73 (t, *J* = 5.9 Hz, 2H), 4.77 (t, *J* = 7.7 Hz, 2H), 2.86 (dd, *J* = 12.5, 6.3 Hz, 4H), 2.09 (t, *J* = 7.9 Hz, 4H), 1.48–1.11 (m, 26H). ^13^C NMR (75 MHz, DMSO-*d*_6_) *δ* 193.31, 170.38, 166.66, 155.57, 141.81, 136.17, 134.20, 131.41, 124.02, 123.92, 77.31, 52.10, 29.02, 28.22, 27.80, 27.73, 23.14. ESI-MS: *m*/*z* calculated for: [M − H]^−^ 777.2989, found: 777.3006. Elemental analysis calcd (%) for C_39_H_46_N_4_O_13_: C 60.15, H 5.95, N 7.19; found: C 60.32, H 6.13, N 7.27.

#### BTDI-Cys

^1^H NMR (600 MHz, DMSO-*d*_6_) *δ* 13.57 (s, 2H), 8.27–8.20 (m, 3H), 8.17–8.09 (m, 3H), 7.29 (dd, *J* = 9.2, 5.9 Hz, 30H), 4.62 (dd, *J* = 11.0, 5.0 Hz, 2H), 3.15–3.08 (m, 2H), 2.98 (dd, *J* = 14.1, 5.0 Hz, 2H). ^13^C NMR (151 MHz, DMSO) *δ* 193.14, 168.78, 166.10, 143.96, 143.77, 129.07, 129.05, 128.33, 128.13, 127.81, 127.51, 126.92, 126.23, 66.66, 55.85, 30.46. ESI-MS: *m*/*z* calculated for: [M − H]^−^ 1011.2415, found: 1011.2435. Elemental analysis calcd (%) for C_61_H_44_N_2_O_9_S_2_: C 72.32, H 4.38, N 2.77; found: C 72.51, H 4.51, N 2.71.

## Conclusions

In conclusion, the use of microwave heating and an appropriate choice of solvent/base pair provide an efficient means of preparing a diverse family of symmetrical diimides based on three structurally distinct aromatic cores and five different amino acids. The developed methodology shows that the syntheses are equally efficient under pressure (small scale) or in open vessels (large scale). The reactions proceed without racemization at α-centre and led to enantiomerically clean products. Short reaction times and facile workup make the developed protocol extremely useful, the present work having provided a library of functionalised diimides with a variety of possible applications.

## Conflicts of interest

There are no conflicts to declare.

## Supplementary Material

RA-008-C8RA05835K-s001
