# Supplementary material for: Highly efficient one-step microwave-assisted synthesis of structurally diverse bis-substituted α-amino acid derived diimides
Source: RSC Adv. 2018 Aug 23;8(52):29840–6. doi: 10.1039/c8ra05835k (PMC9085299; doi:10.1039/c8ra05835k)
Supplement: RA-008-C8RA05835K-s001 [file RA-008-C8RA05835K-s001.pdf]

Electronic Supplementary Material (ESI) for RSC Advances.
This journal is © The Royal Society of Chemistry 2018

Electronic Supplementary Information (ESI)

Highly efficient one-step microwave-assisted synthesis of structurally diverse bis-substituted α-amino acid derived diimides

Marcin Konopka[§a, b], Grzegorz Markiewicz [§a, b] and Artur R. Stefankiewicz*[a, b]

[a] Faculty of Chemistry, Adam Mickiewicz University, ul. Umultowska 89b, 61-614 Poznań, Poland.

[b] Center for Advanced Technologies, Adam Mickiewicz University, ul. Umultowska 89c, 61-614 Poznań, Poland.

[§] These authors contributed equally to this work.

Table of contents

1. ^{1}H and ^{13}C NMR Spectra 2

1.1 PMIs 2

1.2 BPDIs 7

1.3 BTDIs 12

1. ^{1}H and ^{13}C NMR Spectra

1.1 PMIs

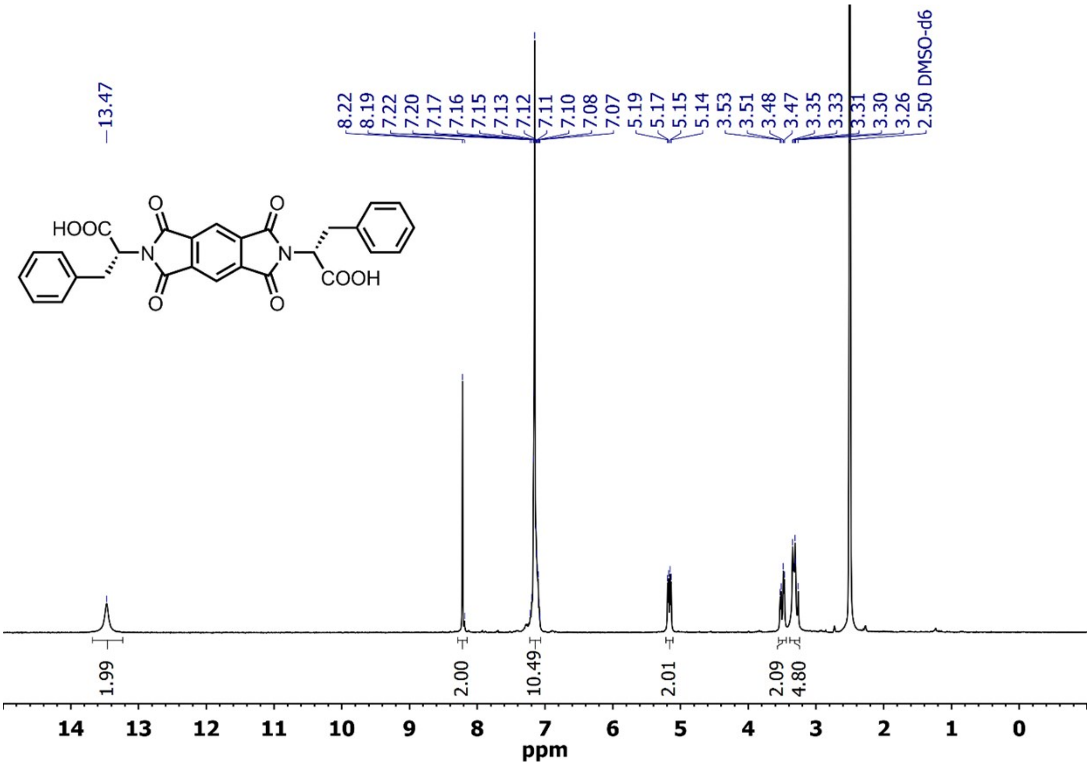

Figure S1. ^{1}H NMR (300 MHz DMSO *d-6*) spectrum of PMI-Phe.

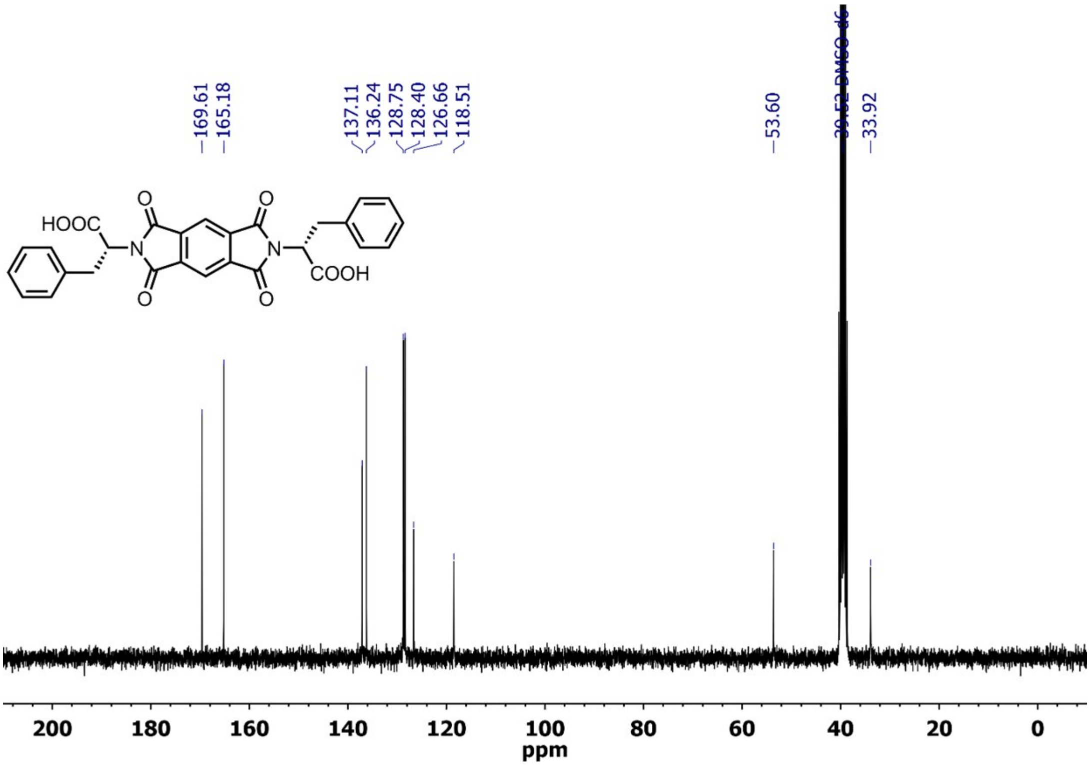

Figure S2. ^{13}C NMR (75 MHz DMSO *d-6*) spectrum of PMI-Phe.

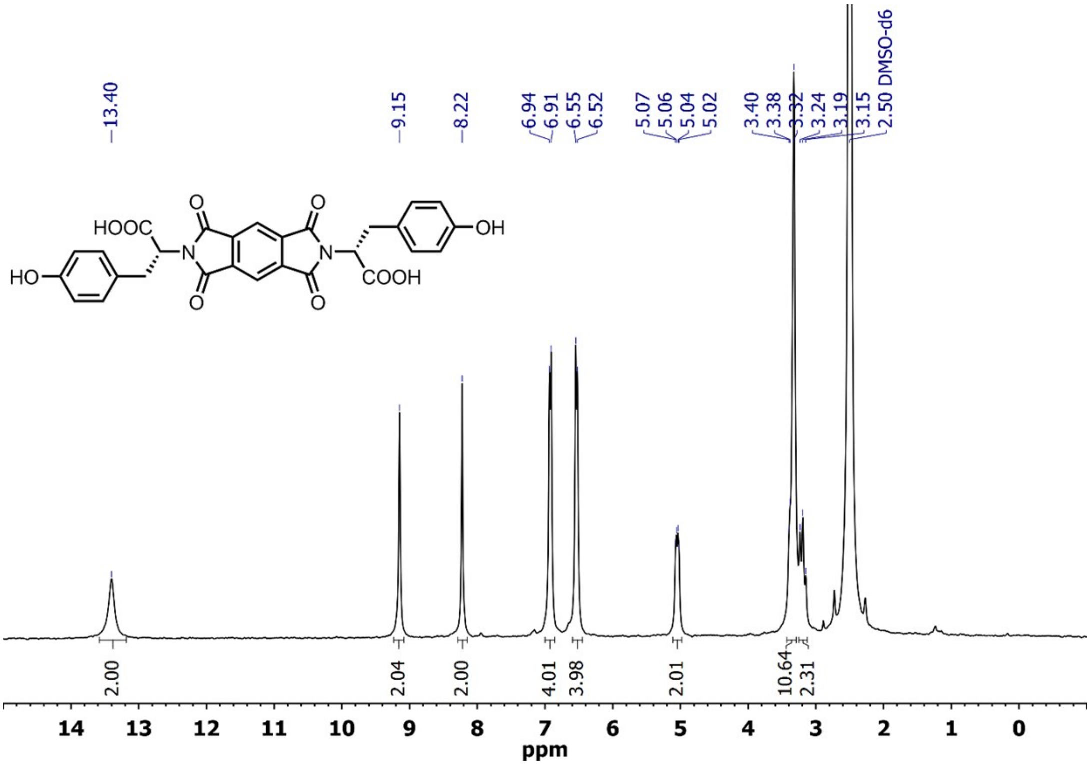

Figure S3. ^{1}H NMR (300 MHz DMSO *d-6*) spectrum of PMI-Tyr.

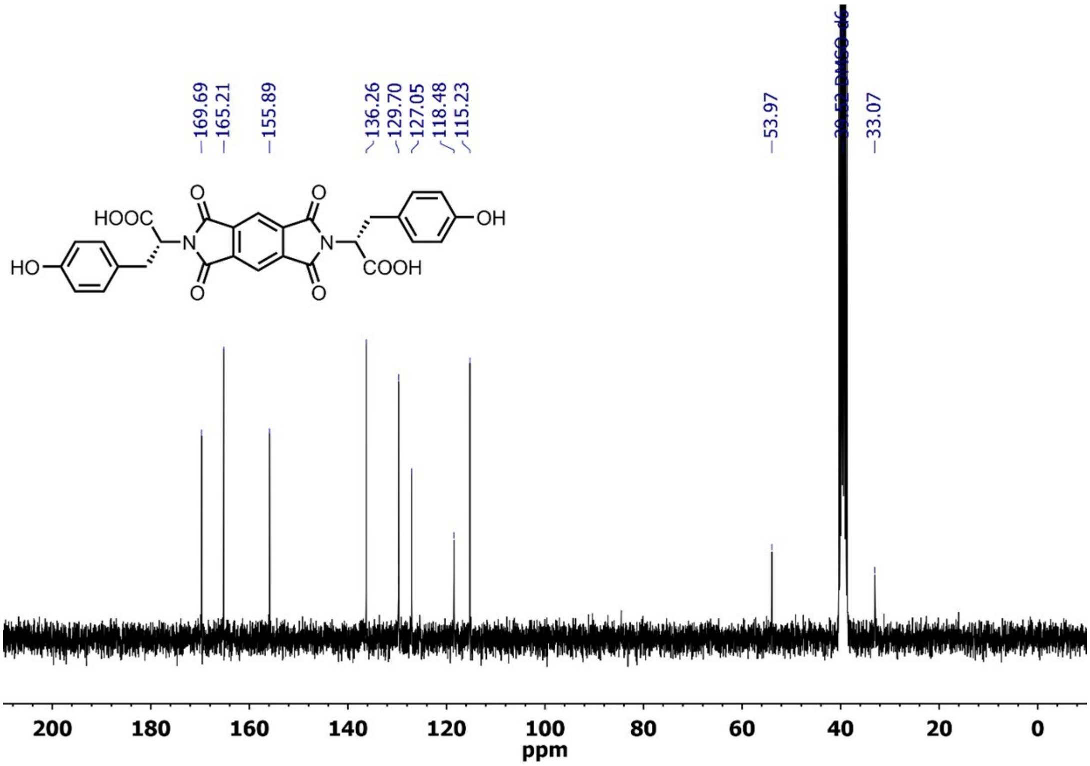

Figure S4. ^{13}C NMR (75 MHz DMSO *d-6*) spectrum of PMI-Tyr.

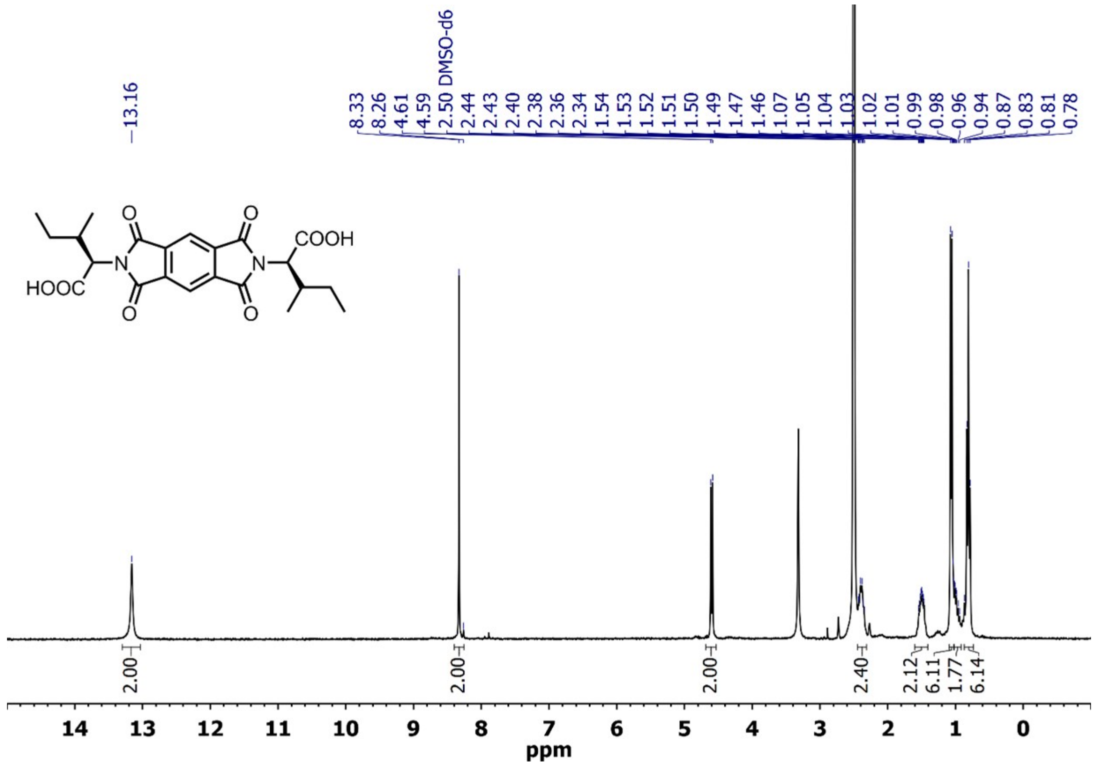

Figure S5. ^{1}H NMR (300 MHz DMSO *d-6*) spectrum of PMI-Ile.

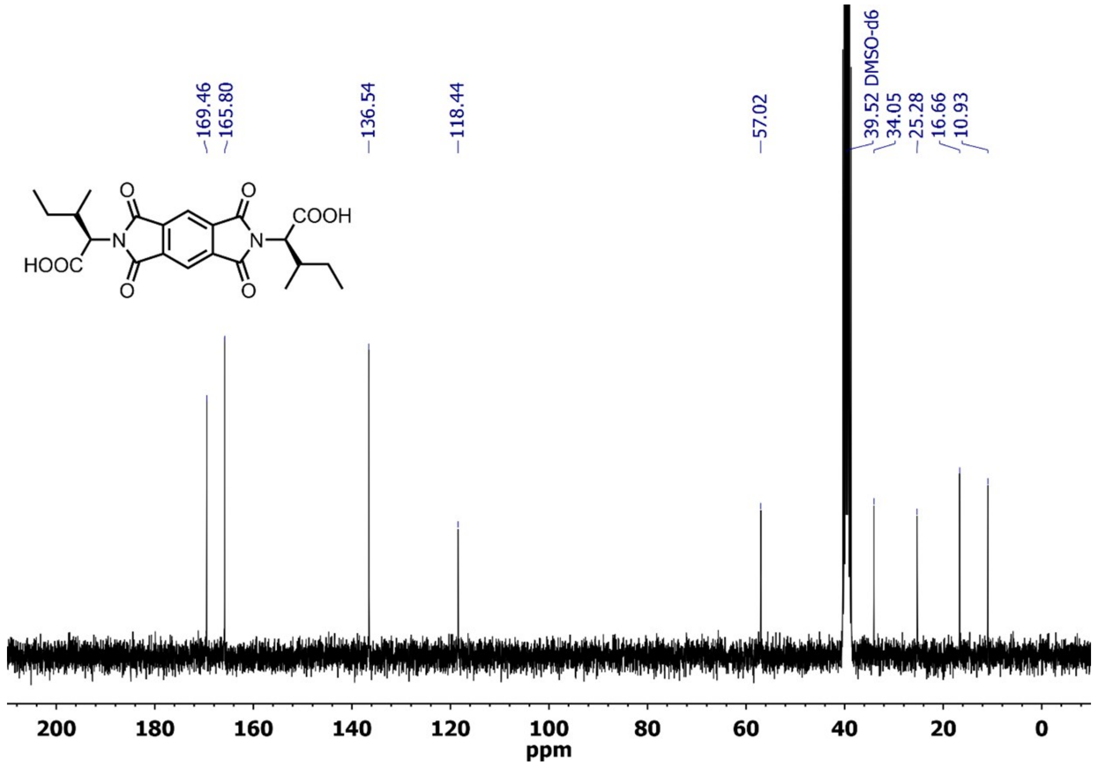

Figure S6. ^{13}C NMR (75 MHz DMSO *d-6*) spectrum of PMI-Ile.

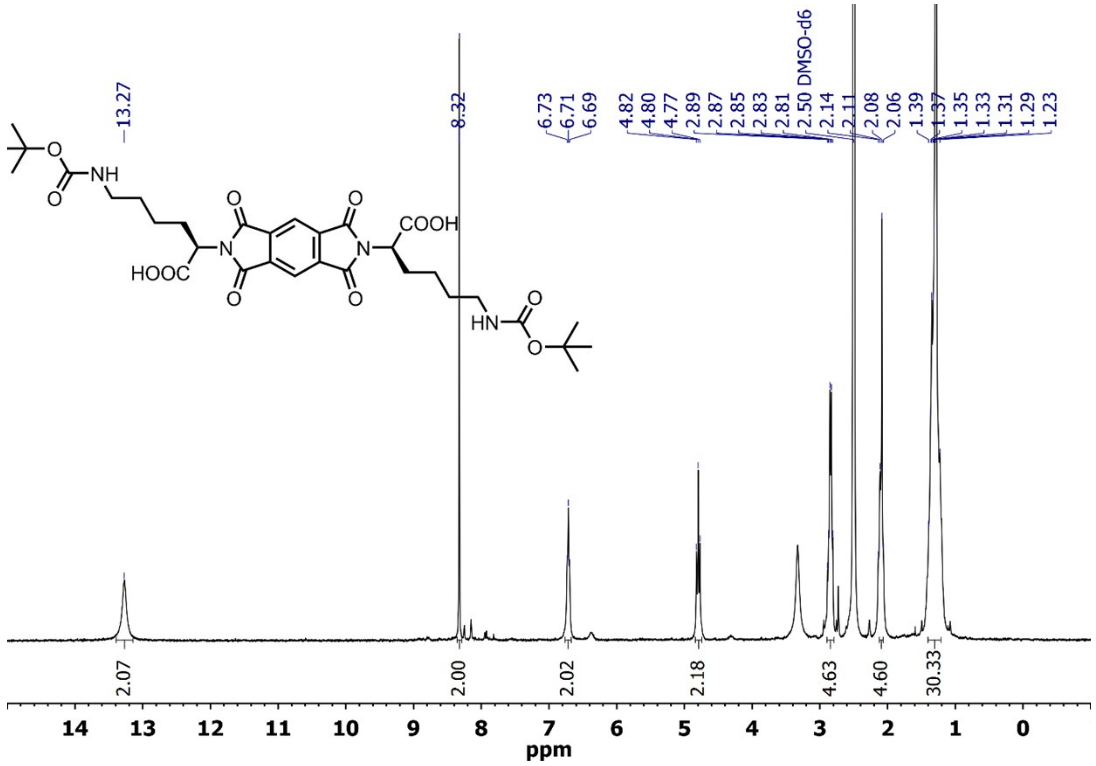

Figure S7. ^{1}H NMR (300 MHz DMSO *d-6*) spectrum of PMI-Lys.

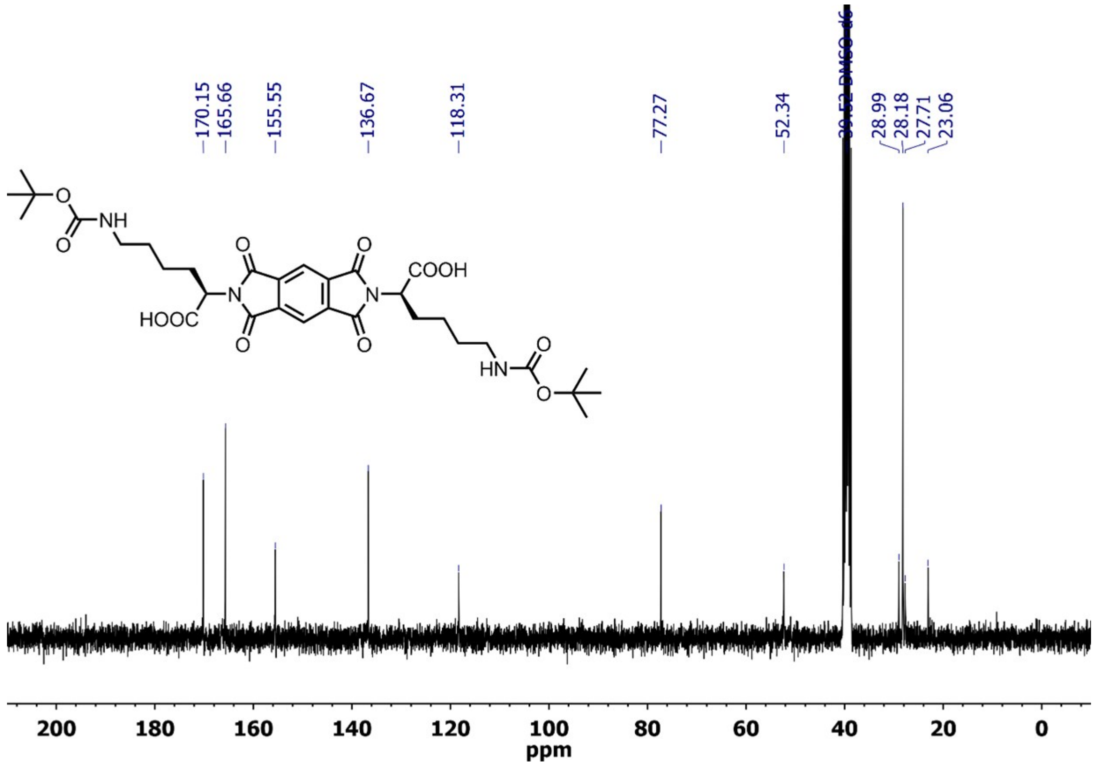

Figure S8. ^{13}C NMR (75 MHz DMSO *d-6*) spectrum of PMI-Lys.

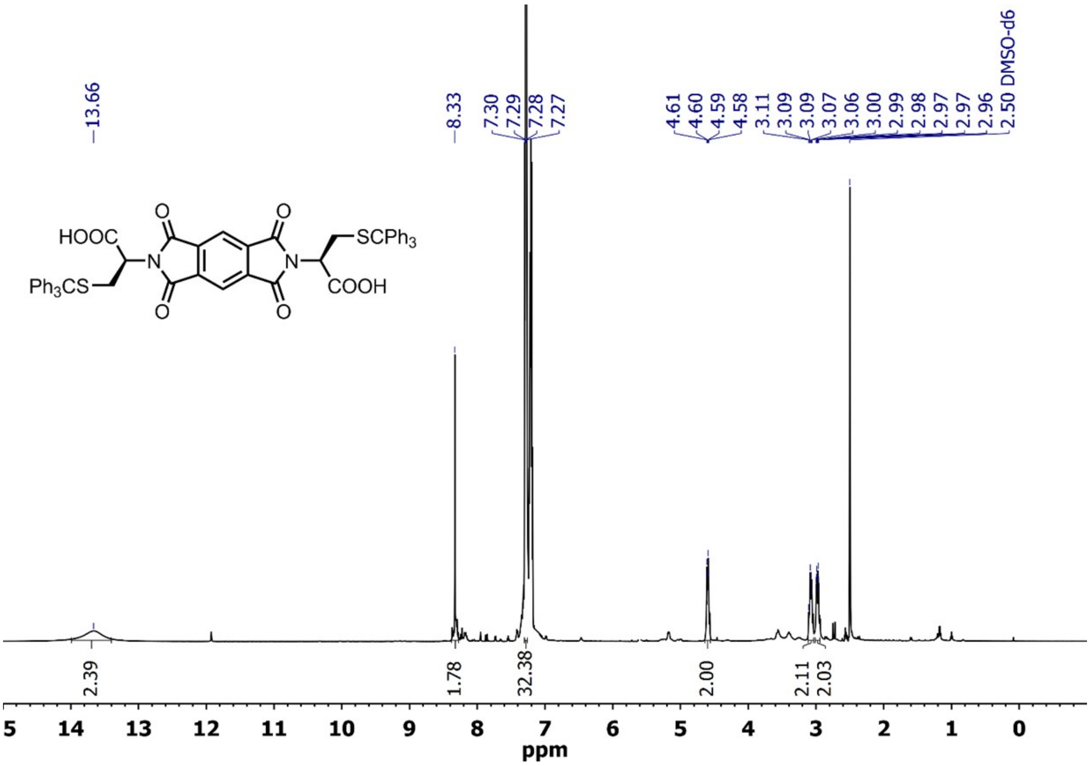

Figure S9. ^{1}H NMR (300 MHz DMSO *d-6*) spectrum of PMI-Cys.

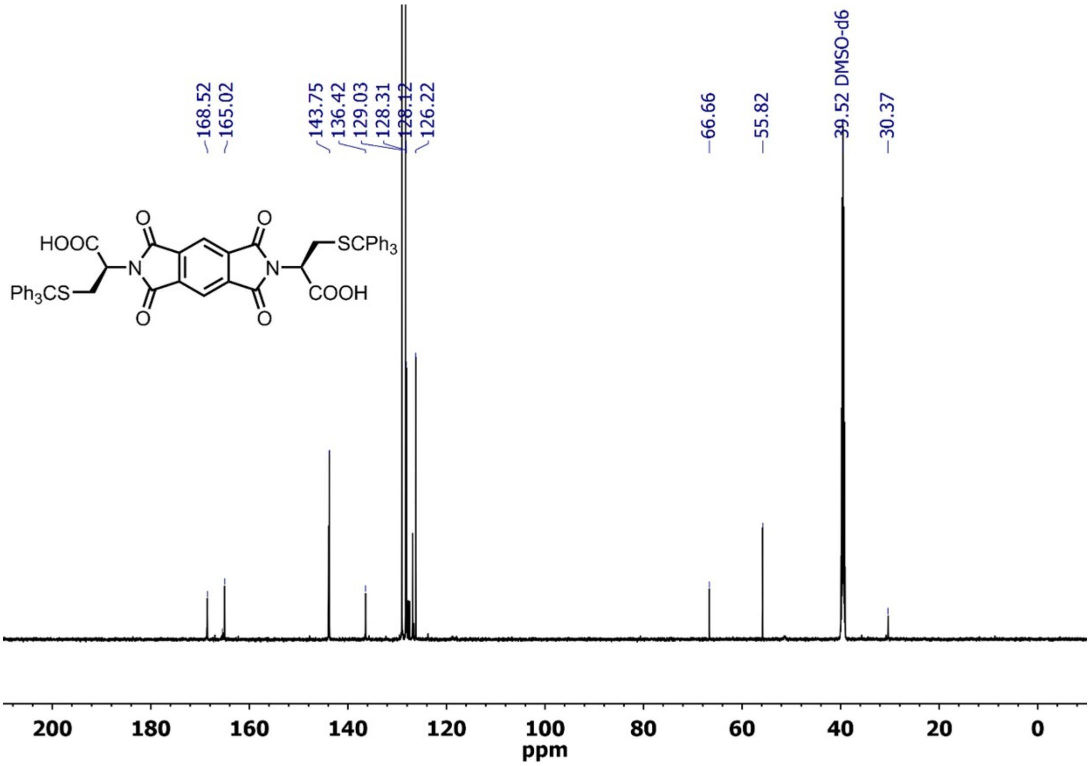

Figure S10. ^{13}C NMR (75 MHz DMSO *d-6*) spectrum of PMI-Cys.

1.2 BPDIs

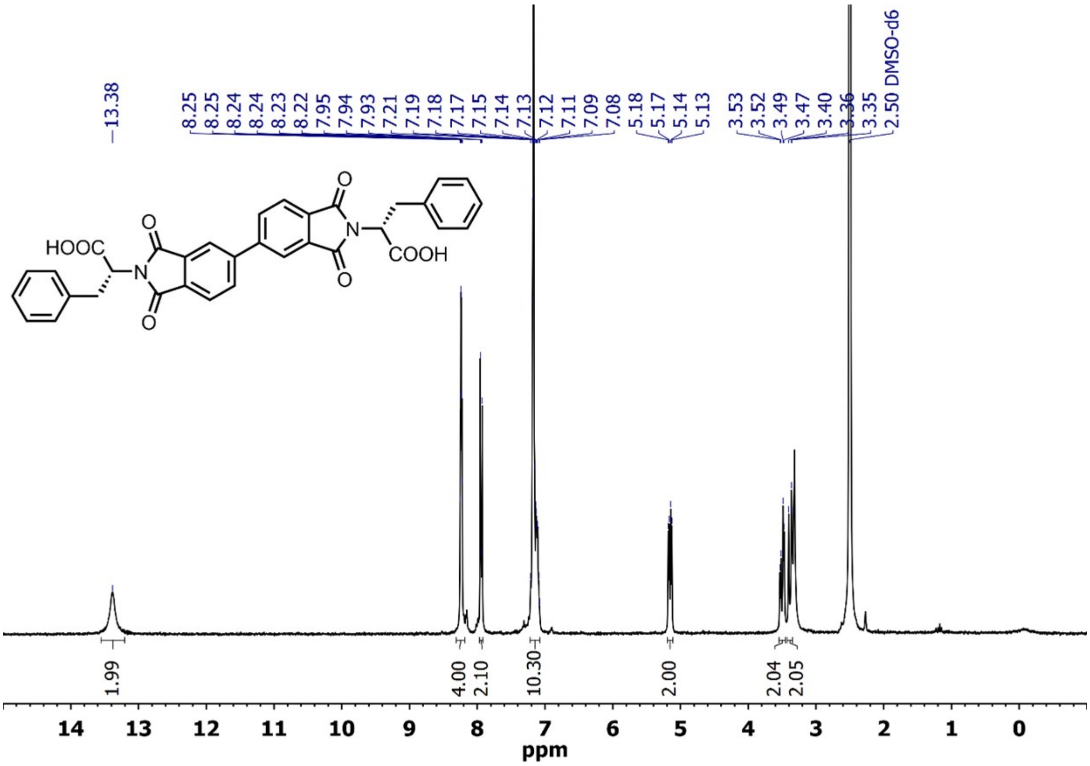

Figure S11. ^{1}H NMR (300 MHz DMSO *d-6*) spectrum of BPDI-Phe.

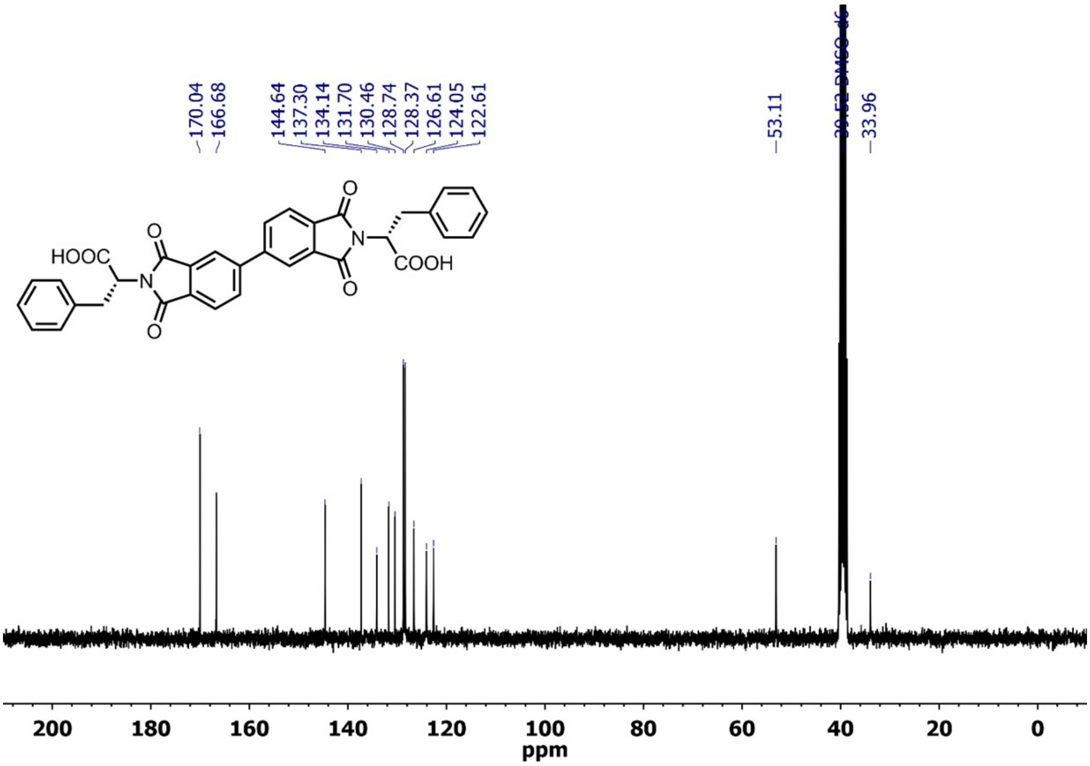

Figure S12. ^{13}C NMR (75 MHz DMSO *d-6*) spectrum of BPDI-Phe.

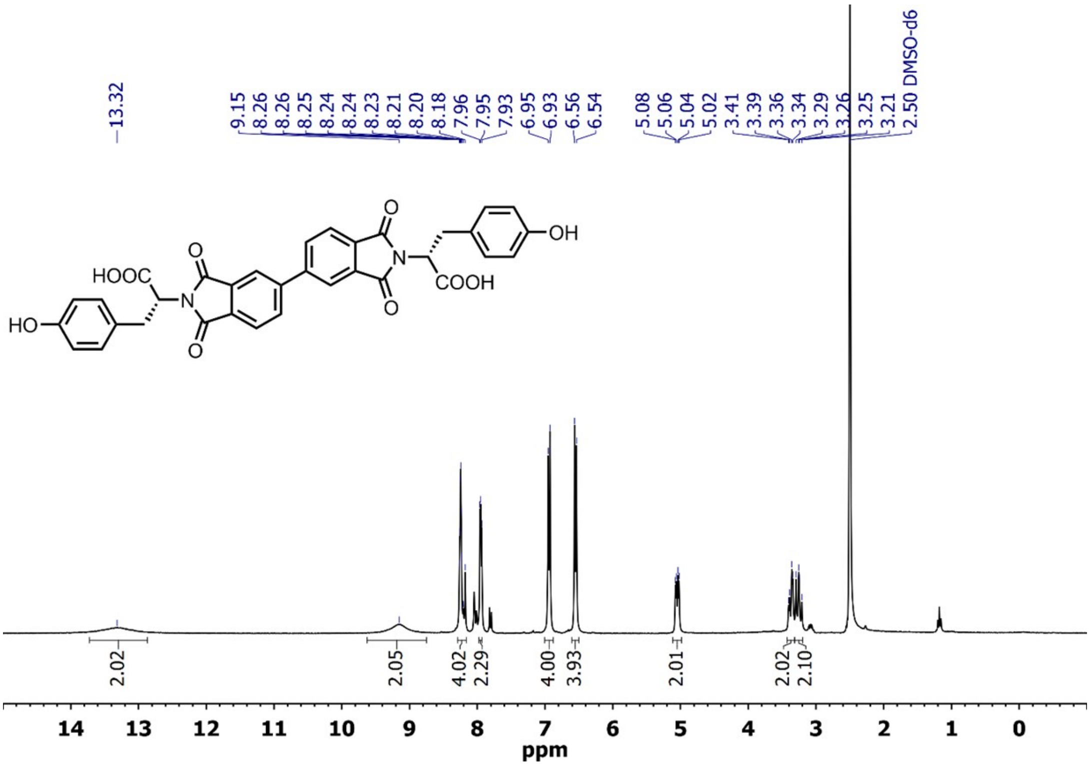

Figure S13. ^{1}H NMR (300 MHz DMSO *d-6*) spectrum of BPDI-Tyr.

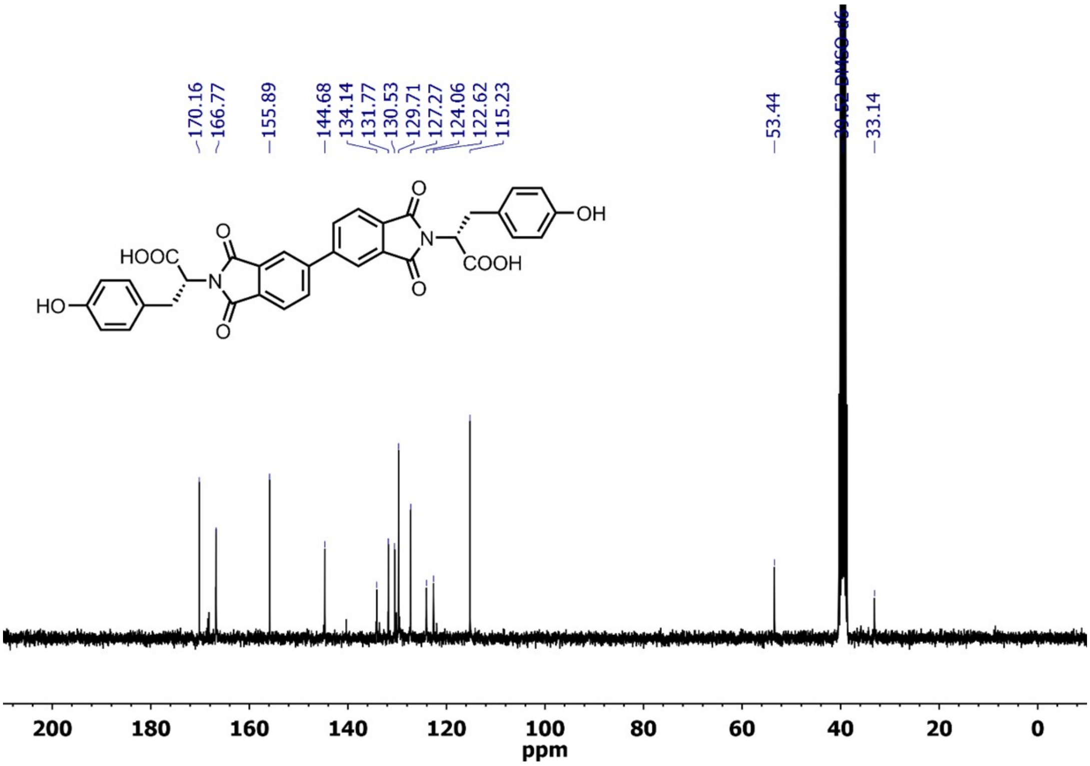

Figure S14. ^{13}C NMR (75 MHz DMSO *d-6*) spectrum of BPDI-Tyr.

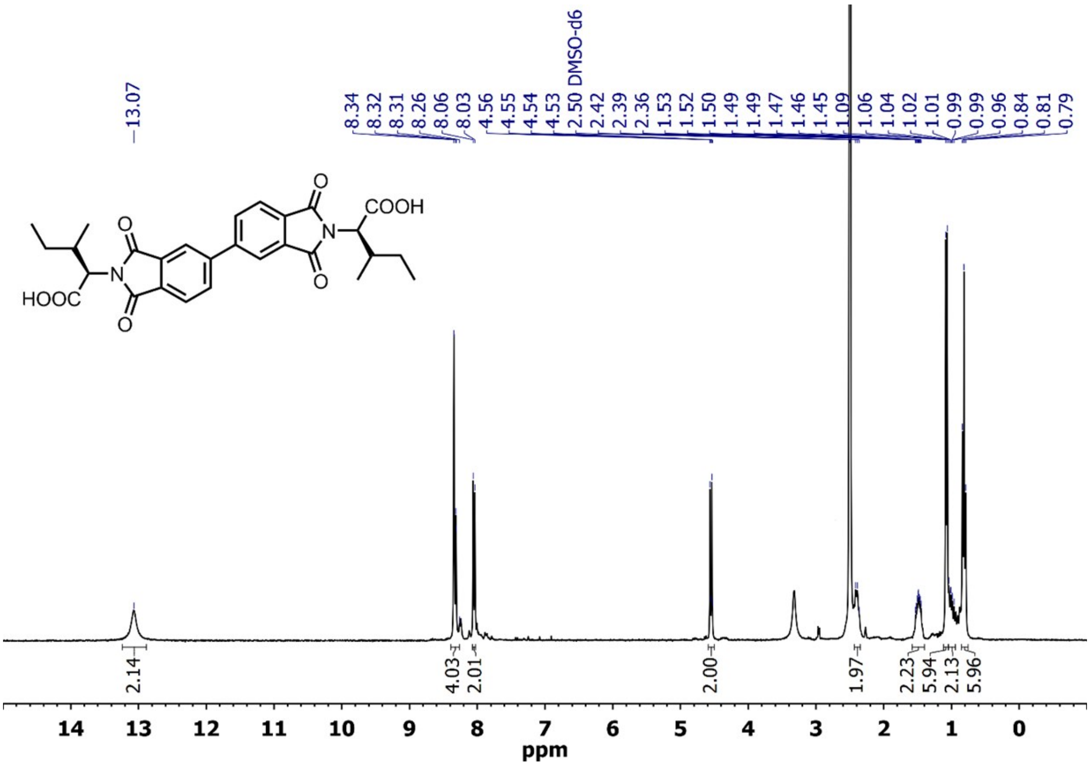

Figure S15. ^{1}H NMR (300 MHz DMSO *d-6*) spectrum of BPDI-Ile.

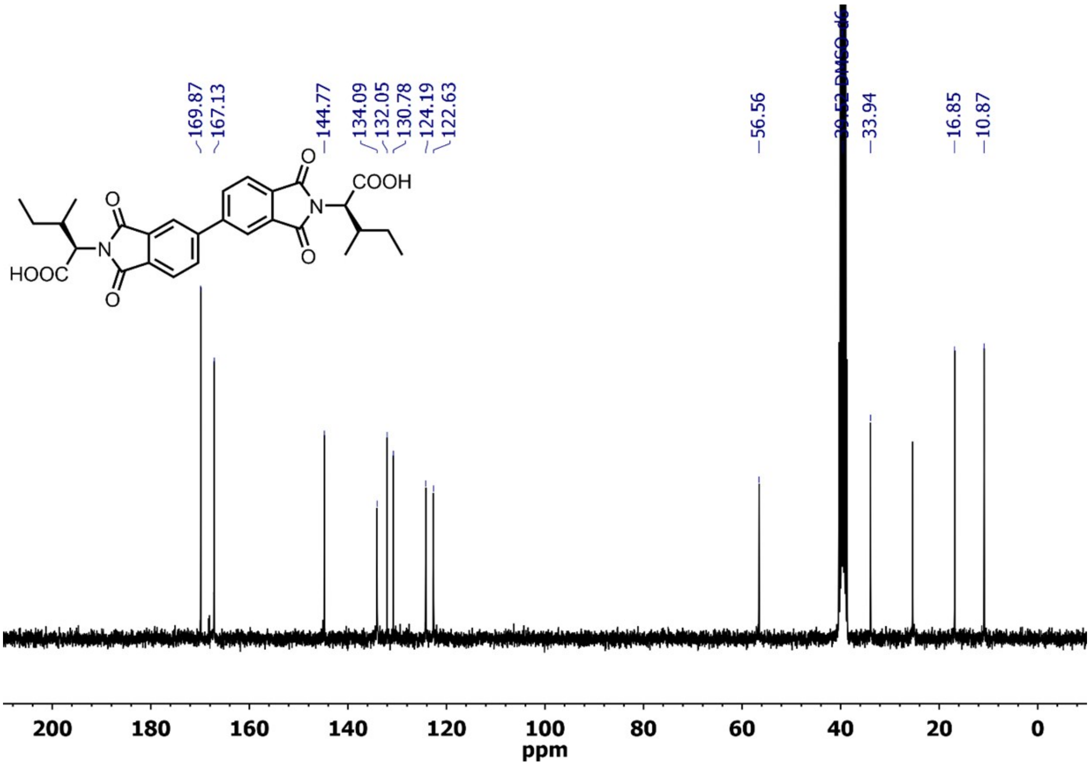

Figure S16. ^{13}C NMR (75 MHz DMSO *d-6*) spectrum of BPDI-Ile.

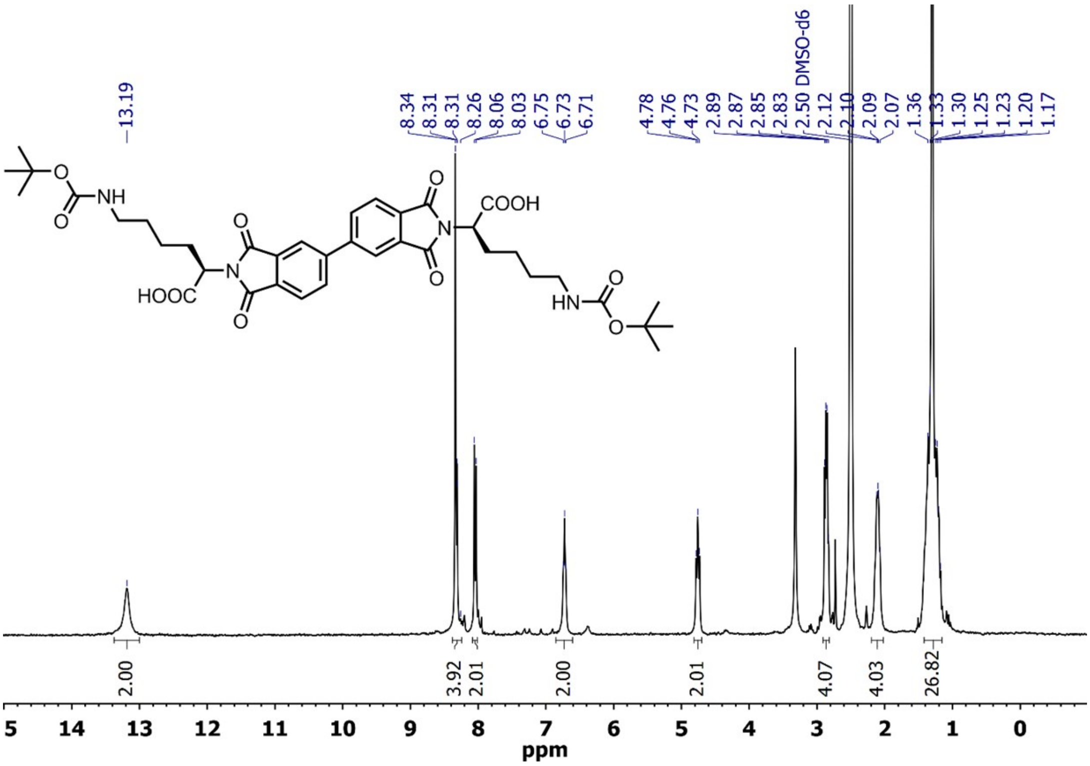

Figure S17. ^{1}H NMR (300 MHz DMSO *d-6*) spectrum of BPDI-Lys.

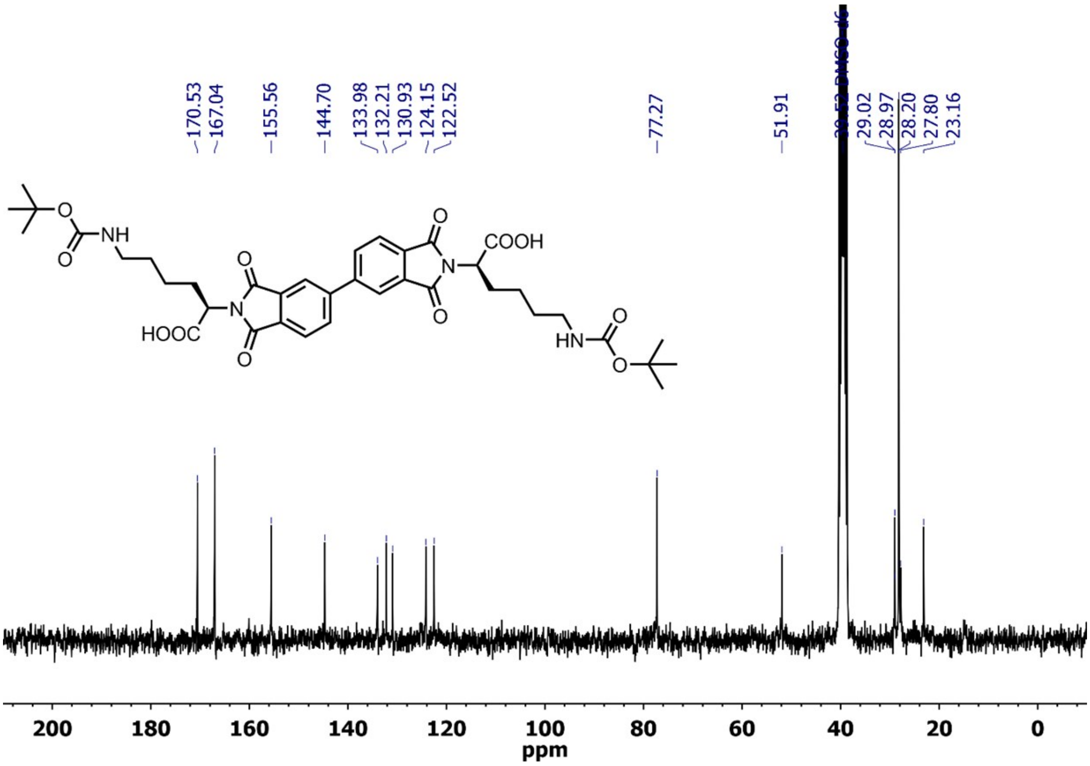

Figure S18. ^{13}C NMR (75 MHz DMSO *d-6*) spectrum of BPDI-Lys.

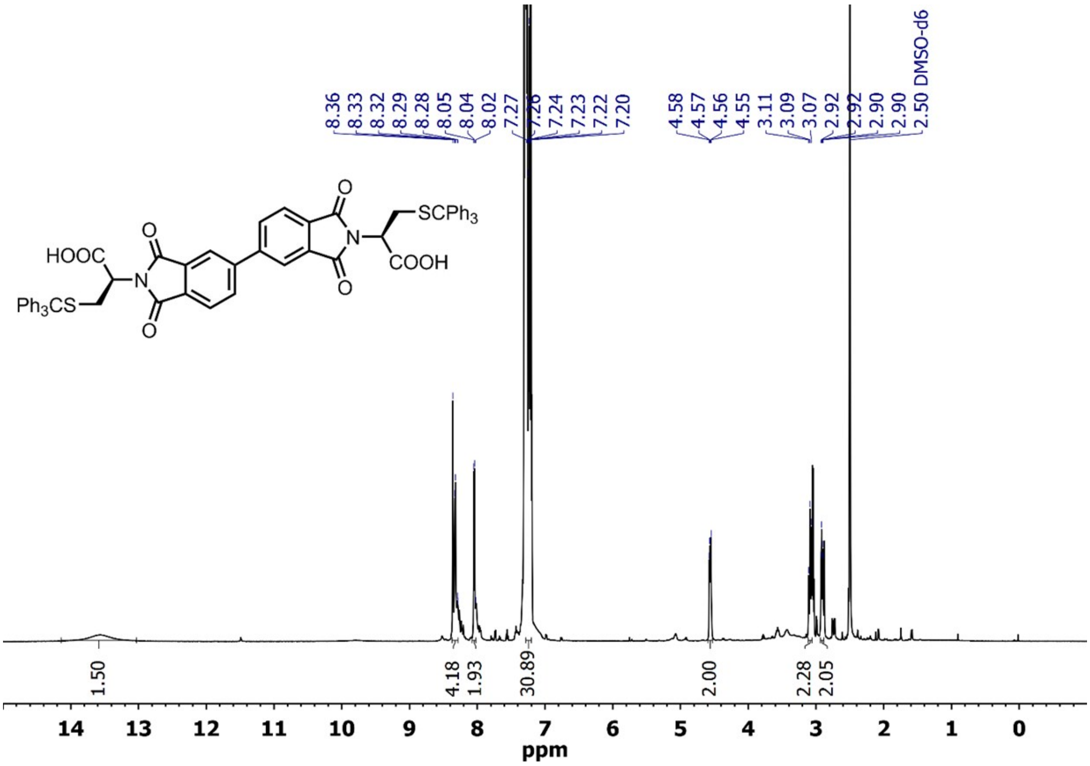

Figure S19. ^{1}H NMR (300 MHz DMSO *d-6*) spectrum of BPDI-Cys.

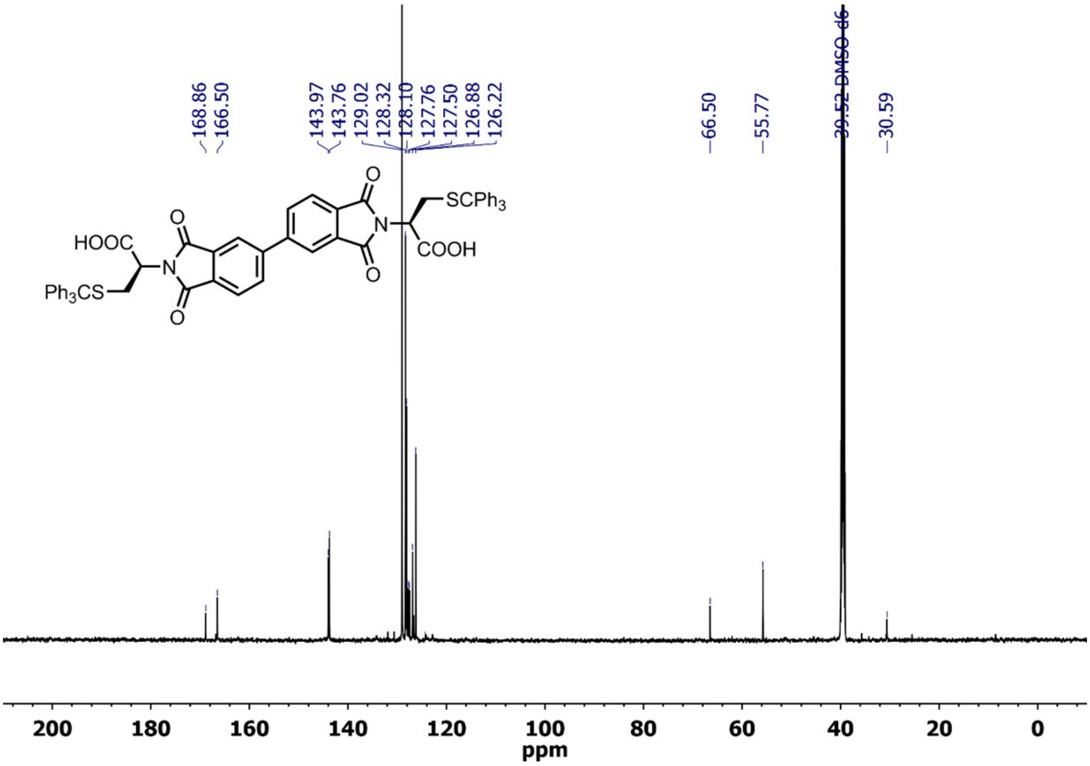

Figure S20. ^{13}C NMR (75 MHz DMSO *d-6*) spectrum of BPDI-Cys.

1.3 BTDIs

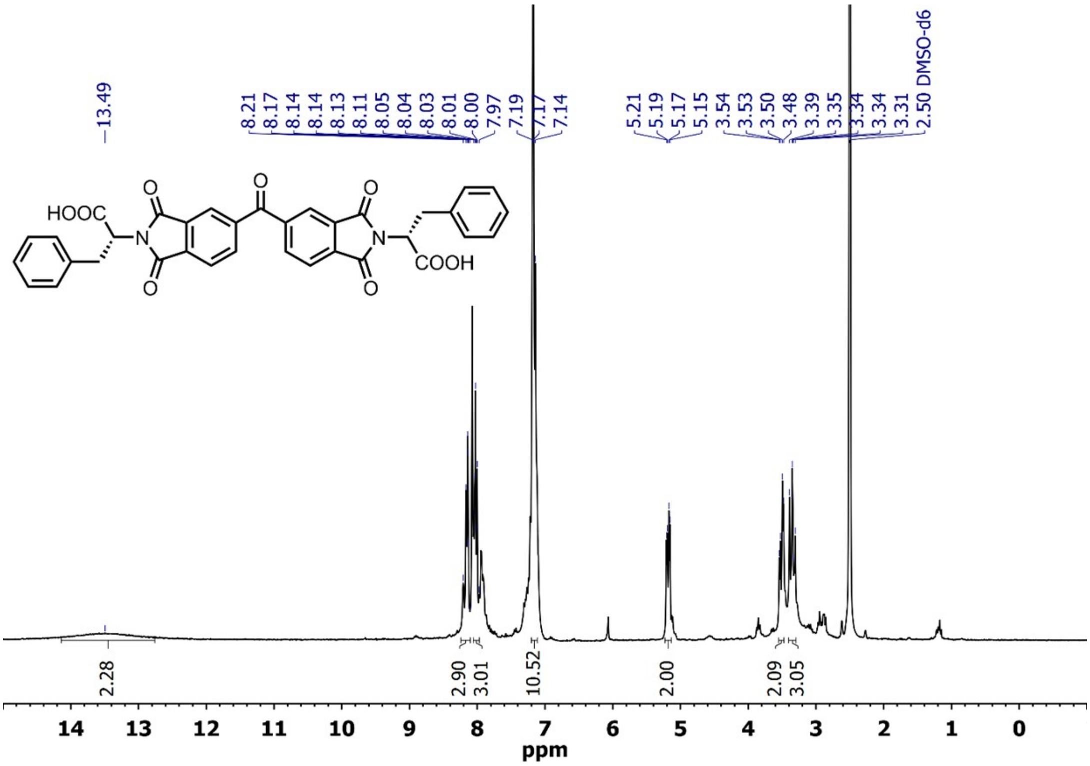

Figure S21. ^{1}H NMR (300 MHz DMSO *d-6*) spectrum of BTDI-Phe.

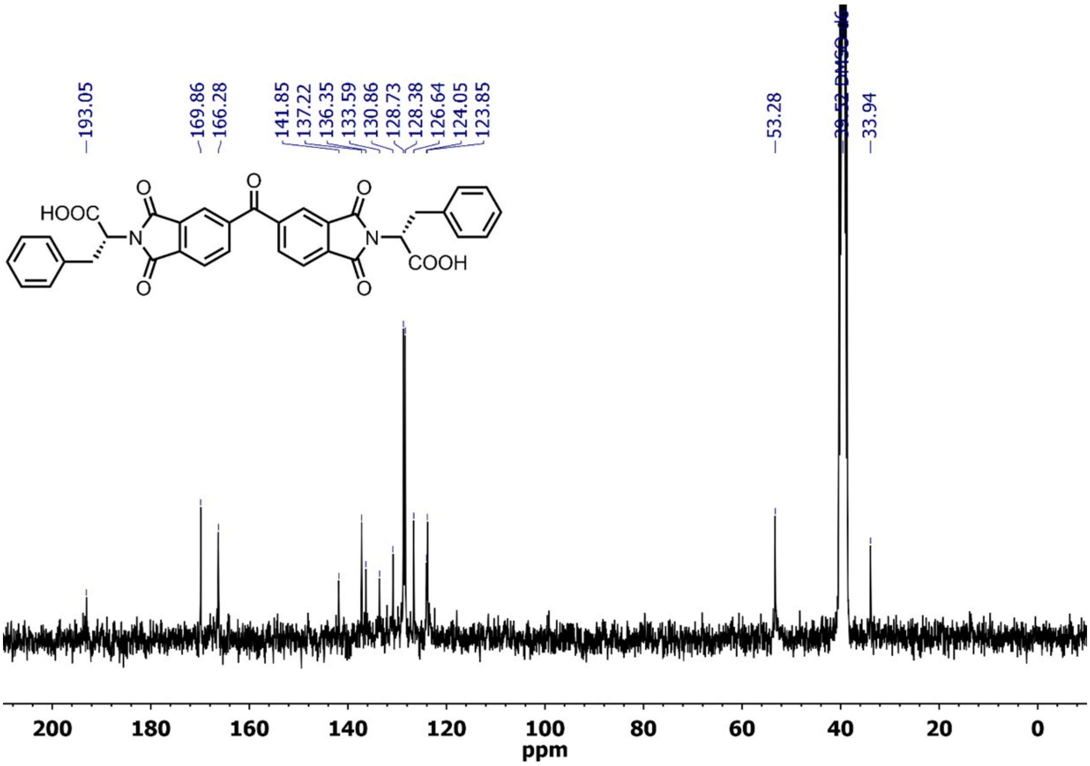

Figure S22. ^{13}C NMR (75 MHz DMSO *d-6*) spectrum of BTDI-Phe.

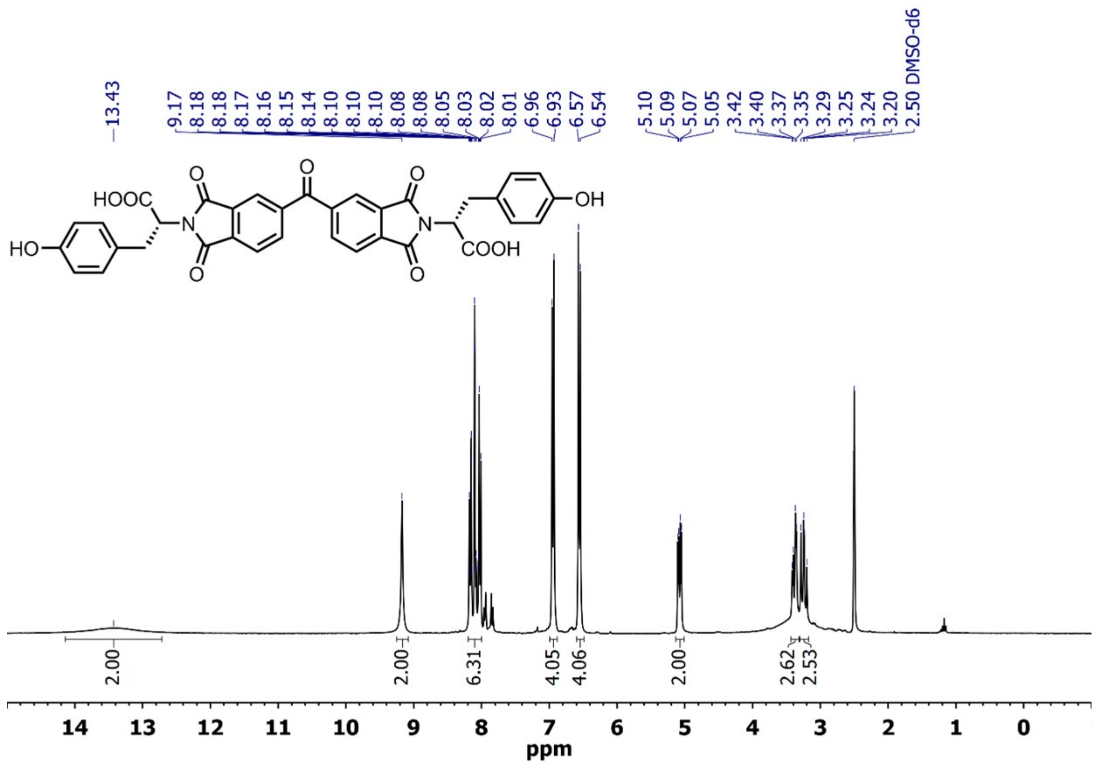

Figure S23. ^{1}H NMR (300 MHz DMSO *d-6*) spectrum of BTDI-Tyr.

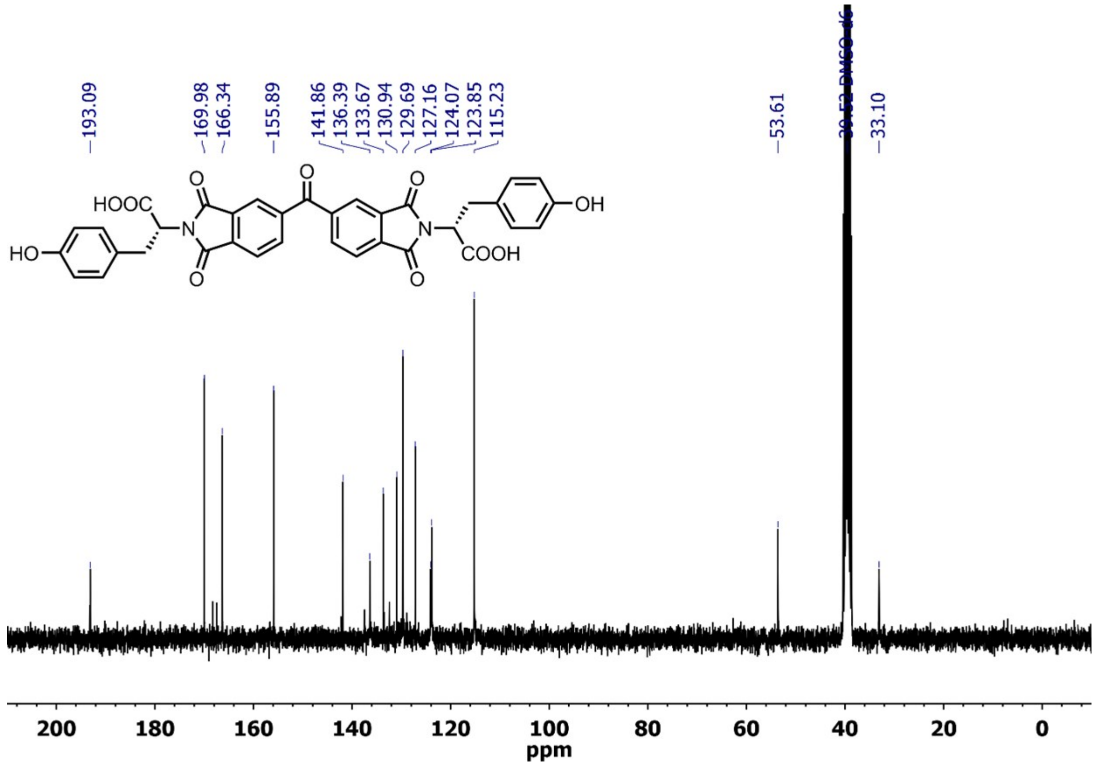

Figure S24. ^{13}C NMR (75 MHz DMSO *d-6*) spectrum of BTDI-Tyr.

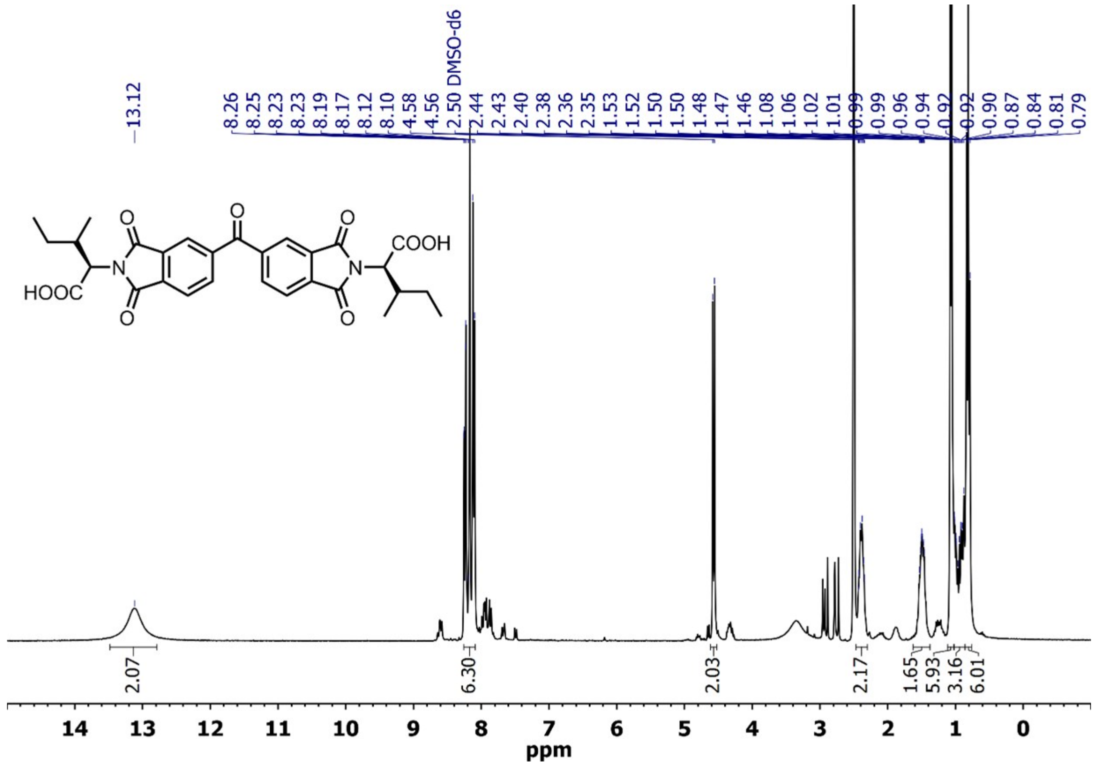

Figure S25. ^{1}H NMR (300 MHz DMSO *d-6*) spectrum of BPDI-Ile.

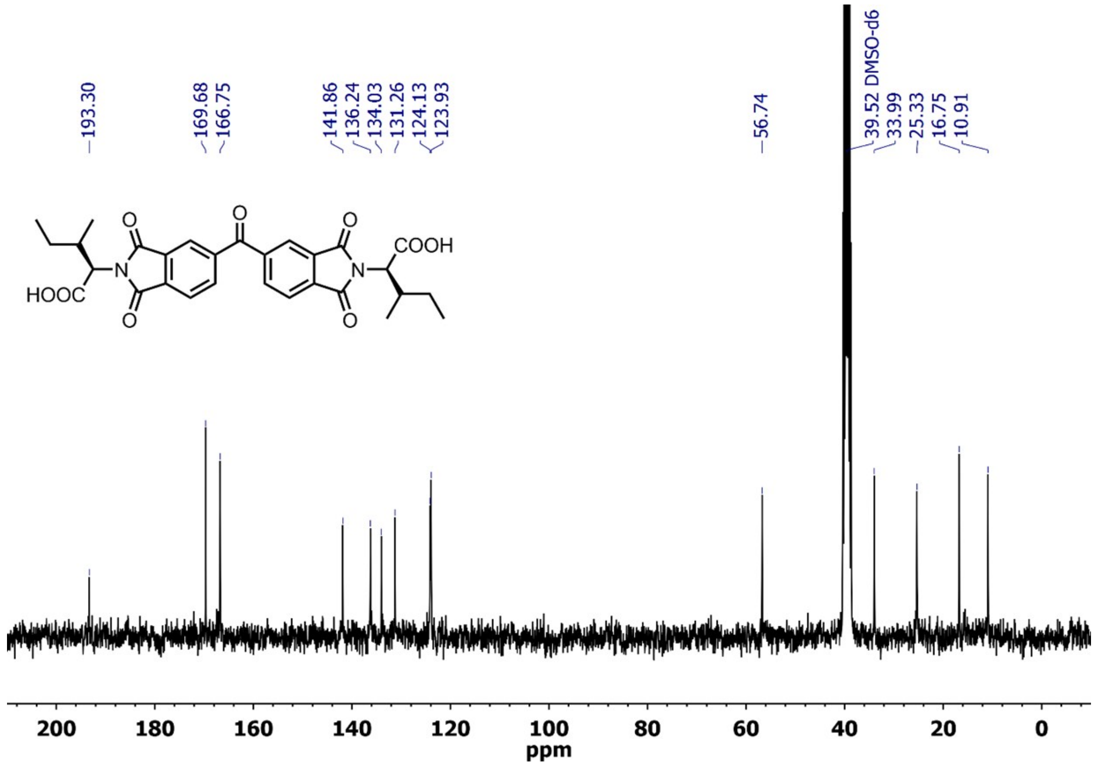

Figure S26. ^{13}C NMR (75 MHz DMSO *d-6*) spectrum of BTDI-Ile.

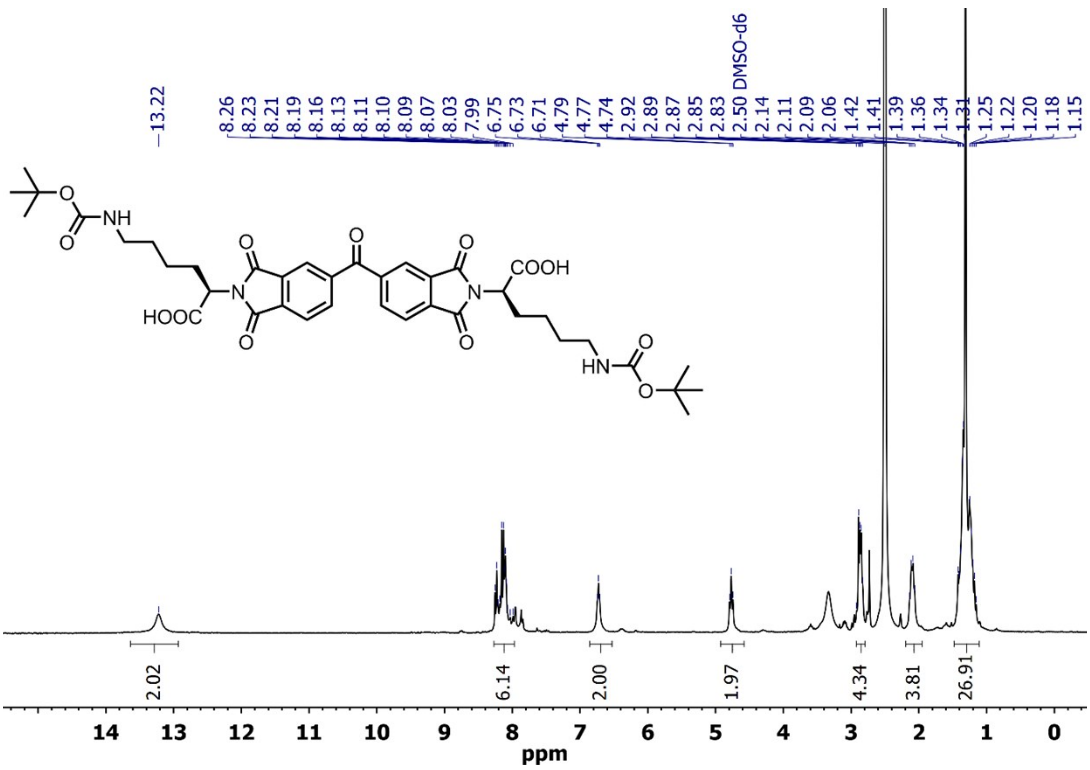

Figure S27. ^{1}H NMR (300 MHz DMSO *d-6*) spectrum of BTDI-Lys.

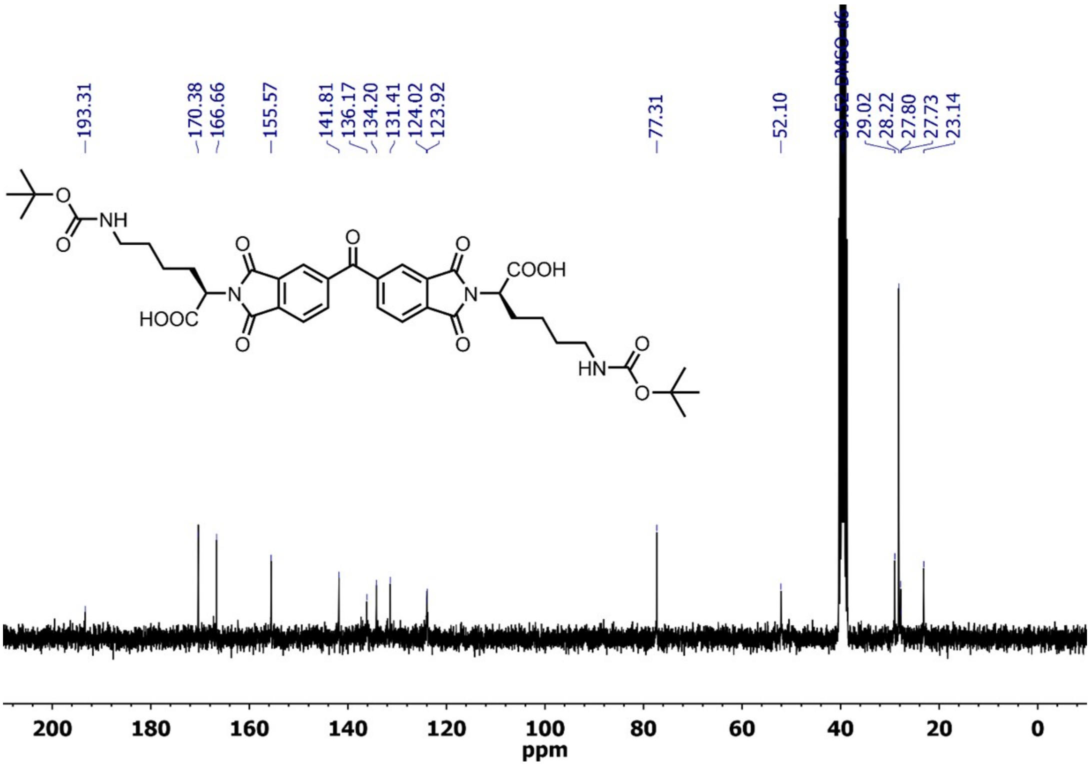

Figure S28. ^{13}C NMR (75 MHz DMSO *d-6*) spectrum of BTDI-Lys.

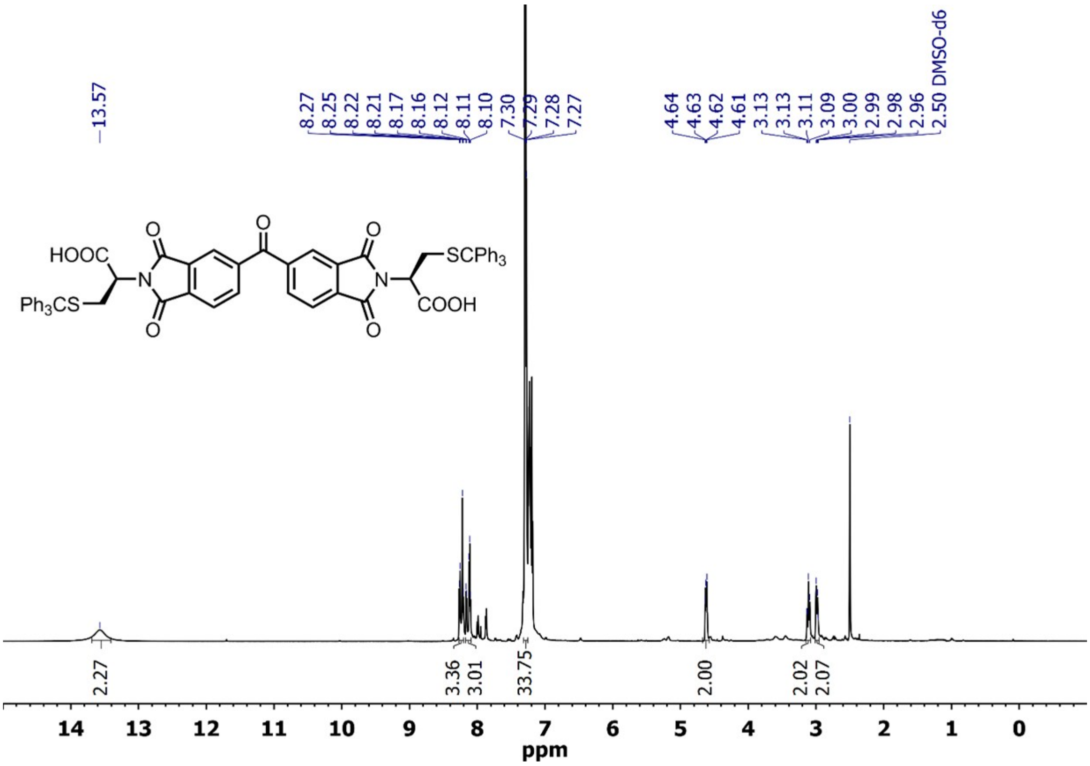

Figure S29. ^{1}H NMR (300 MHz DMSO *d-6*) spectrum of BTDI-Cys.

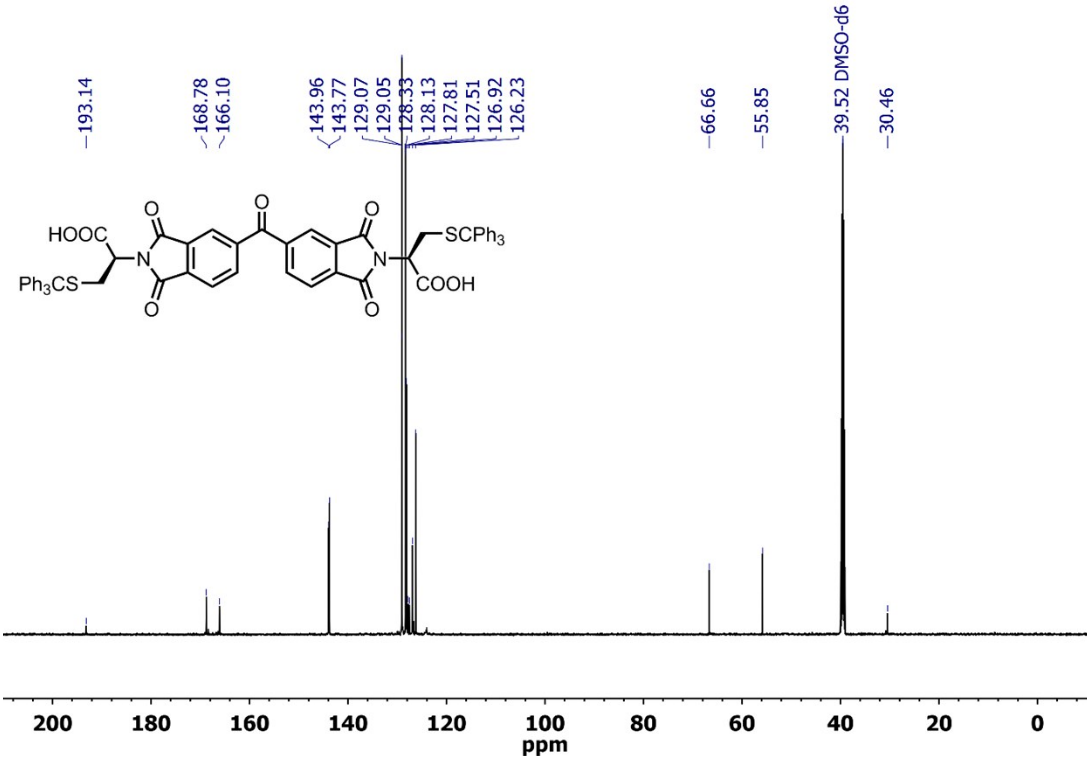

Figure S30. ^{13}C NMR (75 MHz DMSO *d-6*) spectrum of BTDI-Cys.
